# Effects of a biotechnologically produced *Pleurotus sapidus* mycelium on gut microbiome, liver transcriptome and plasma metabolome of broilers

**DOI:** 10.1016/j.psj.2024.103975

**Published:** 2024-06-13

**Authors:** Lea Schäfer, Sarah M. Grundmann, Martin Rühl, Holger Zorn, Waldemar Seel, Marie-Christine Simon, Sven Schuchardt, Erika Most, Robert Ringseis, Klaus Eder

**Affiliations:** ⁎ Institute of Animal Nutrition and Nutrition Physiology, Justus Liebig University Giessen, Giessen, Germany; † Institute of Food Chemistry and Food Biotechnology, Justus Liebig University Giessen, Giessen, Germany; ‡ Fraunhofer Institute for Molecular Biology and Applied Ecology IME, Giessen, Germany; § University of Bonn, Nutrition and Microbiota, Institute of Nutrition and Food Sciences, Bonn, Germany; # Fraunhofer Institute for Toxicology and Experimental Medicine ITEM, Hannover, Germany; ǁ Center for Sustainable Food Systems, Justus Liebig University Giessen, Giessen, Germany

**Keywords:** broiler, liver transcriptome, plasma metabolome, gut microbiome, mycelium

## Abstract

Submerged cultivation using low-value agro-industrial side streams allows large-scale and efficient production of fungal mycelia, which has a high nutritional value. As the dietary properties of fungal mycelia in poultry are largely unknown, the present study aimed to investigate the effect of feeding a *Pleurotus sapidus* (**PSA**) mycelium as a feed supplement on growth performance, composition of the cecal microbiota and several physiological traits including gut integrity, nutrient digestibility, liver lipids, liver transcriptome and plasma metabolome in broilers. 72 males, 1-day-old Cobb 500 broilers were randomly assigned to 3 different groups and fed 3 different adequate diets containing either 0% (PSA-0), 2.5% (PSA-2.5) and 5% (PSA-5.0) *P. sapidus* mycelium in a 3-phase feeding system for 35 d. Each group consisted of 6 cages (replicates) with 4 broilers/cage. Body weight gain, feed intake and feed:gain ratio and apparent ileal digestibility of crude protein, ether extract and amino acids were not different between groups. Metagenomic analysis of the cecal microbiota revealed no differences between groups, except that one α-diversity metric (Shannon index) and the abundance of 2 low-abundance bacterial taxa (Clostridia UCG 014, Eubacteriales) differed between groups (*P* < 0.05). Concentrations of total and individual short-chain fatty acids in the cecal digesta and concentrations of plasma lipopolysaccharide and mRNA levels of proinflammatory genes, tight-junction proteins, and mucins in the cecum mucosa did not differ between groups. None of the plasma metabolites analyzed using targeted-metabolomics differed across the groups. Hepatic transcript profiling revealed a total of 144 transcripts to be differentially expressed between group PSA-5.0 and group PSA-0 but none of these genes was regulated greater 2-fold. Considering either the lack of effects or the very weak effects of feeding the *P. sapidus* mycelium in the broilers it can be concluded that inclusion of a sustainably produced fungal mycelium in broiler diets at the expense of other feed components has no negative consequences on broilers´ performance and metabolism.

## INTRODUCTION

According to the latest estimates, the world population will increase from 7.9 billion people in 2022 to 8.6 billion people in 2032. The rise in world population will result in a global increase in the consumption of animal products which is associated with an increased requirement for animal feed (OECD/FAO, 2023). The strongest increase in animal production will be in poultry meat that is affordable compared to other livestock meat and has a favorable combination of a high protein and a low lipid concentration. However, feed production is not only complicated by the fact that natural resources such as farmland and water are becoming increasingly limited but also that the production of feed for poultry as a monogastric species competes with human nutrition (Wu et al., 2014). It is therefore of great importance to produce animal feed with increased sustainability compared to conventionally produced feed using feedstuffs which do not compete with human nutrition.

Agricultural side streams with a high fiber content fulfill these demands, because they are used in human nutrition only to a limited extent. In monogastric animals, such side streams with a high fiber content, like brewer's grain, sugar beet pulp and pomace, have also a very limited nutritional value due to their low digestibility (Huang and Lee, 2018). However, a potential strategy to increase the nutritional value of side streams with a low digestibility is to use them as a substrate for the production of edible mushrooms. Mushrooms possess a unique enzyme system enabling them to degrade a wide array of non-starch polysaccharide- and lignin-containing substrates contained in the abovementioned side streams and, thereby, to upcycle low-value side streams into high-value fungal biomass. The most commonly cultivated mushrooms include *Agaricus bisporus, Lentinula* and *Pleurotus spp.* (Chang and Buswell, 1996). Mushrooms have a high nutritional value due to their high concentrations of protein, fiber, vitamins and minerals, in combination with a low concentration of lipids (Mattila et al., 2001; Valverde et al., 2015). While commercial mushroom products are obtained from fruiting bodies, which are produced in a time-exhausting process, submerged cultivation has the potential to produce mycelial biomass at large-scale and in an efficient manner (Papaspyridi et al., 2012). Apart from providing protein, fiber, vitamins and minerals, the mycelium of *Pleurotus spp.* could be interesting for poultry nutrition due to its high concentration of β-glucans. The β-glucans are well-known to act as potent prebiotics in monogastric animals (Lam and Chi-Keung Cheung, 2013). Prebiotics beneficially modulate the structure of the gut microbial community through promoting the growth of beneficial commensal bacteria, such as *Lactobacillus* spp. and *Bifidobacterium* spp.. The commensals protect the outer mucin layer from colonization by opportunistic pathogens, thereby, strengthening the intestinal barrier function, which strongly relies on tight-junction proteins, for example, claudins (**CLDN**) and occludin (**OCLN**), sealing the space between adjacent intestinal epithelial cells and different mucin (**MUC**) glycoproteins, for example, MUC2 and MUC5AC, preventing the direct bacterial contact with the intestinal epithelial cells. Apart from modulating the gut microbiota structure, prebiotics have been shown to exert several beneficial metabolic effects including blood and liver lipid-lowering and anti-inflammatory effects (Cheung, 1998; Manzi, 2000; Maheshwari et al., 2020). The latter effects are supposed to be mediated by several gut-derived compounds, such as short-chain fatty acids (**SCFA**), and the improved gut barrier function, which prevents the translocation of pro-inflammatory bacterial components, for example, lipopolysaccharide (**LPS**), into the systemic circulation.

As the dietary properties of fungal mycelia in poultry feeding are largely unknown, the present study aimed to investigate the effect of feeding *P. sapidus* mycelium as a dietary supplement, included into the diet at the expense of wheat, on growth performance, composition of the cecal microbiota and several physiological traits including gut integrity, nutrient digestibility, liver lipids, liver transcriptome and plasma metabolome in broilers. For this end, several screening technologies, such as transcriptomics, metabolomics, and metagenomic analysis of the gut microbiota were applied. The hypothesis of the study was that the β-glucans, which have been documented to exert a prebiotic effect, in the *P. sapidus* mycelium have beneficial effects on the gut microbial community and influence gut integrity, metabolic health and performance in a favorable manner.

## MATERIAL AND METHODS

### Biotechnological Production of *P. sapidus* Mycelium

*P. sapidus* (German Collection of Microorganisms and Cell Cultures DSMZ, strain number 8266) was used for the production of vegetative mycelium. All cultivation procedures were performed in a sterile environment. The chemicals utilized were purchased from Carl Roth (Karlsruhe, Germany) and Sigma-Aldrich (Merck, Darmstadt, Germany). The stock culture was cultivated according to the method of Ahlborn et al. (2019). A mixture of 30 g/L malt extract and 3 g peptone was used for the precultures. After a 6-d cultivation period at 24°C in the dark on a rotary shaker at 110 rpm (KS 4000 i control, IKA, Staufen, Germany), the preculture was transferred to a main culture. The medium of the main culture contained yeast extract, magnesium sulfate hydrate, potassium dihydrogen phosphate, and different trace elements as described by Trapp et al. (2018). In addition, the main culture media contained isomaltulose molasses (**ISM**) from Südzucker AG (Offstein, Germany). After another 6-d cultivation period at 24°C in the darkness on a rotary shaker, the main culture was homogenized using Art-Miccra D-8 (ART Labortechnik, Müllheim, Germany) and transferred to a 150 L bioreactor (Biostat D 100, B. Braun International, Melsungen, Germany) for fermentation (Maheshwari et al., 2020). The bioreactor was equipped with a Rushton-type impeller, a pH electrode, and a temperature sensor. The main culture was grown at 24°C and 150 rpm with an air-flow rate of 3.0 NL per min. After 6 d, *P. sapidus* mycelium was harvested using a 250-micron sieve, thoroughly washed with water, freeze-dried, and stored at -20°C (Maheshwari et al., 2020).

### Chemical Analysis of *P. sapidus* Mycelium

Crude protein (**CP**), crude ash (**CA**), ether extract (**EE**), crude fiber (**CF**), sugar, starch, fatty acids and amino acids in the *P. sapidus* mycelium were analyzed according to the official methods (Verband Deutscher Landwirtschaftlicher Untersuchungs- und Forschungsanstalten, VDLUFA, 2012). For the measurement of glucans, the enzyme assay kit for mushroom and yeast β-glucan from Megazyme (Ireland) was used in accordance with the manufacturer's protocol. The chitin content was determined by a colorimetric assay with modifications described recently (Ahlborn et al., 2019; Maheshwari et al., 2020).

### Animals, Experimental Design, and Diets

The 5-wk feeding trial with 72 males, 1-day-old broiler chickens (Cobb 500, Cobb-Vantress, Weidemar, Germany) was approved by the Animal Welfare Officer of the Justus Liebig University Giessen (approval no.: JLU 843_M). All experimental procedures described followed established guidelines for laboratory animals care and handling. The broilers were randomly assigned to 3 different groups (4 broilers/cage, 6 cages/group), with a similar mean initial body weight (38.1 ± 2.9 g; mean ± SD; *N* = 72) across the groups. The broilers were housed in 2.1 m^2^ cages with nipple drinkers and feed automates and had free access to feed and water. The floor was covered with hemp-based litter (Hemparada, HempFlax Group B.V., Oude Pekela, Netherlands) which allowed scratching, pecking and dustbathing and was exchanged 2 times per week during the first 2 wk and every 2 d during the last 3 wk of the trial. In addition, broilers were provided with perches in elevated position for resting and sleeping. Light regime followed a schedule of 24 h:0 h, 23 h:1 h, 22 h:2 h, 21 h:3 h, 20 h:4 h, 19 h:5 h (light:dark) at d 1, 2, 3, 4, 5, 6, and 18 h:6 h from d 7 onward and the light intensity was constantly 40 Lux, as recommended by the breeder. The room temperature decreased from 28 to 29°C on d 1, measured at pen height, to 23 to 24°C on d 35. Infrared lamps (Albert Kerbl GmbH, Buchbach, Germany) were used as additional heat sources during the first 6 d to adjust the temperature at the cage floor to 34°C. Mean relative humidity was 60.0 ± 1.9%. The groups were fed 3 different nutrient adequate diets containing either 0% (group PSA-0), 2.5% (group PSA-2.5) or 5% (group PSA-5.0) *P. sapidus* mycelium in a 3-phase feeding system (starter diet form d 1 to 10, grower diet from d 11 to 21, finisher diet from d 22 to 35). The composition of the 3 diets is shown in Table 1. The composition of the diets met the broiler's requirements of nutrients and energy according to the breeder's recommendations (Cobb-Vantress, 2022). Diets were provided in crumbled form during the first 3 d, and in pellet form (2 mm diameter) from d 3 until the end of the trial. On d 1, 10, 21, and 35 body weight (individually) and feed intake (per cage) were determined. The feed:gain ratio was calculated from feed intake and body weight gains on cage basis.

**Table 1:** Composition of the broiler diets supplemented with 0% (PSA-0), 2.5% (PSA-2.5) or 5% (PSA-5.0) of *Pleurotus sapidus* mycelium.

	Starter diets			Grower diets			Finisher diets		
	PSA-0	PSA-2.5	PSA-5.0	PSA-0	PSA-2.5	PSA-5.0	PSA-0	PSA-2.5	PSA-5.0
Components (g/kg)									
Maize	283.7	281.9	284	280	280	280	320	320	320
Soybean meal (42% CP)	373	370	370	310	310	310	270	270	270
Wheat	203.7	193.7	176.7	268.7	254.6	240	265.6	251	236.6
*P. sapidus* mycelium	0	25	50	0	25	50	0	25	50
Soybean oil	50	50	50	50	50	50	50	50	50
Cellulose	20	10	0	20	10	0	20	10	0
Mineral & vitamin mix^1^	20	20	20	20	20	20	20	20	20
Monocalcium phosphate	15	15	15	15	15	15	15	15	15
Calcium carbonate	15.5	15.5	15.5	15.5	15.5	15.5	15.5	15.5	15.5
Sodium chloride	4	4	4	4	4	4	4	4	4
DL-Methionine	3.8	3.8	3.8	3.6	3.6	3.6	3.4	3.4	3.3
L-Lysine	4.4	4.3	4.2	4.6	4.5	4.3	4.4	4.2	4.1
L-Threonine	2	2	2	1.8	1.7	1.6	1.5	1.5	1.4
L-Arginine	1.9	1.8	1.8	2	1.9	1.8	1.8	1.7	1.6
L-Valine	2	2	2	3	2.7	2.7	2.2	2.2	2.1
L-Isoleucine	1	1	1	1.8	1.5	1.5	1.6	1.5	1.5
Titanium dioxide	0	0	0	0	0	0	5	5	5

1 The mineral & vitamin mix supplied the following minerals and vitamins per kg diet (starter/grower/finisher): Fe, 40/40/40 mg; Cu, 15/15/15 mg; Mn, 100/100/100 mg; Zn, 100/100/100 mg; I, 1/1/1 mg; Se, 0.35/0.35/0.35 mg; vitamin A, 10,000/10,000/10,000 IU; vitamin D3, 5,000/5,000/5,000 IU; vitamin K3, 3/3/3 mg; vitamin E, 80/50/50 IU; vitamin B1, 3/2/2 mg; vitamin B2, 9/8/6 mg; vitamin B6, 4/3/3 mg; vitamin B12, 0.02/0.015/0.015 mg; biotin, 0.2/0.18/0.18 mg; folic acid, 2/2/1.5 mg; nicotinic acid, 60/50/50 mg; choline chloride, 500/400/350 mg; pantothenic acid, 15/12/10 mg.

### Sample Collection

On d 35, all animals were killed by bleeding (opening of *Vena jugularis* and *Arteria carotis*) under electrical anesthesia using a BTG-40A stunning device (Westerhoff Geflügeltechnik, Hoogstede, Germany) in accordance with the European legislation for euthanasia of animals (EUR_Lex, 09.01.2024). For determination of the metabolic parameters twelve broilers per group (2 broilers from each cage), whose body weight represented the mean values of the whole group, were selected. Whole blood was individually collected into tubes containing ethylenediaminetetraacetic acid (9 mL S-Monovette, Sarsted, Numbrecht, Germany) as an anti-coagulant. For plasma preparation, whole blood was centrifuged (1,100 × *g*, 10 min) at 4°C and stored at -80°C for further analysis. The liver was excised, weighted and aliquots were snap-frozen in liquid nitrogen and stored at -80°C until analysis. The gastrointestinal tract was removed and digesta and mucosa from the ileum and the cecum were collected. Mucosa and digesta samples were snap-frozen in liquid nitrogen and stored at -80°C until analysis. Feed samples were collected after feed manufacturing and stored at -20°C.

### Determination of the Chemical Composition of the Diets

Concentrations of dry matter (**DM**), CP, CA, EE, CF, sugar, starch, amino acids and fatty acids in the main diet components (wheat, maize, soybean extraction meal) and the experimental diets were determined using the official methods (Verband deutscher landwirtschaftlicher Untersuchungs- und Forschungsanstalten, VDLUFA, 2012). To calculate the CP content of the *P. sapidus* mycelium, a specific, chitin corrected *N*-to-protein conversion factor of 4.17 (Souci et al., 2016) was used, for all other components and for the experimental diets a *N*-to-protein conversion factor of 6.25 were used. To calculate the apparent *N*-corrected metabolizable energy (**AME*_N_***) content of the diets, the formula of the World's Poultry Science Association for poultry compound feed was used (WPSA, 1984):

$$\text{AM}E_{N}\left(\text{MJ}/\text{kg}\right)=\left[\left(0.01551*\text{crude}\;\text{protein}\right)+\left(0.03431*\text{crude}\;\text{lipids}\right)+\left(0.01669*\text{starch}\right)+\left(0.01301*\text{sugar}\right)\right]$$

### Determination of Apparent Ileal Digestibility

The determination of the apparent ileal digestibility (**AID)** of EE, CP and amino acids at the end of the experiment was performed with the indicator method using titanium dioxide (**TiO_2_**) as an inert marker in the finisher diets (d 22–35) (Short et al., 1996). Prior to analysis, ileal digesta samples were freeze-dried and manually grounded under nitrogen with a mortar. The concentration of the indigestible indicator TiO_2_ in the ileal digesta was determined according to the method of Brandt and Allam (1987) with slight modifications. Concentrations of EE, CP and amino acids in the ileal digesta were determined by official methods as described above. Based on the ileal concentrations of indicator, the AID of EE, CP and amino acids were calculated according to the following formula:

$$\text{AID}\left(\%\right)=100-\left[\left(\text{Ti}O_{2\_\text{Diet}}/\text{Ti}O_{2\_\text{Digesta}}\right)\times \left(\text{Nutrient}{\_}_{\text{Digesta}}/\text{Nutrient}{\_}_{\text{Diet}}\right)\times 100\right],$$

in which TiO_2_Diet_ is the TiO_2_ concentration in the diet (% DM), TiO_2_Digesta_ is the TiO_2_ concentration in the ileal digesta (% DM), Nutrient__Digesta_ is the nutrient (EE, CP, amino acids) concentration in ileal digesta (% DM), and Nutrient__Diet_ is the nutrient (EE, CP, amino acids) concentration in the diet (% DM).

### Determination of Microbiota Composition and Diversity in the Cecal Digesta

16S rRNA gene amplicon sequencing was performed at Life & Brain as described recently in detail (Beller et al., 2024). QIIME 2 version 2022.8 was used to process the 16S sequencing data (Bolyen et al., 2019). Raw sequencing data were deposited as FASTQ files at the NCBI´s Sequence Read Archive (**SRA**) repository under BioProject accession number PRJNA1120330. DADA2 was deployed for sequencing quality control, including filtering for PhiX reads and chimeric sequences, and denoising (Callahan et al., 2016). To identify amplicon sequencing variants (**ASV**) with >99% similarity, a pretrained SILVA classifier (silva-138-nr99-16S-V3-V4-classifier) was used. The α-diversity, β-diversity, and relative abundance were analyzed with the MicrobiomeAnalyst platform using the default settings (Dhariwal et al., 2017; Chong et al., 2020).

### Determination of SCFA Concentrations in the Cecal Digesta

SCFA concentrations in cecal digesta were determined as previously described (Fiesel et al., 2014). In brief, 50 mg aliquots of cecal digesta were mixed with 0,5 mL 5% o-phosphoric acid containing internal standard (0.15 mg/mL crotonic acid). Extraction was performed by vortexing for 3 min followed by centrifugation at 21,000 × *g* for 10 min at 4°C. One μL of the extract was injected into a gas chromatograph (Clarus 580 GC system, Perkin Elmer, Waltham, MA) equipped with a polar capillary column (30 m free fatty acid phase, 0.32 mm internal diameter, 0.25 μm film thickness; Macherey and Nagel, Düren, Germany) and a flame ionisation detector.

### Concentration of LPS in Plasma

The concentration of LPS in plasma was analyzed using an ELISA Kit (Chicken Lipopolysaccharide ELISA Kit, LOT: 2K235N; Assay Genie, Dublin, Ireland) according to the manufacturer's protocol.

### Total RNA Extraction From Liver and Cecum Mucosa and qPCR Analysis

Aliquots from the liver (20–30 mg) and aliquots from cecum mucosa (30–50 mg) were used for total RNA extraction using TRIzol reagent (Invitrogen, Karlsruhe, Germany) according to the manufacturer´s protocol. Total RNA was analyzed for quantity and quality using an Infinite 200M microplate reader with a NanoQuant plate (both from Tecan, Mainz, Germany). The mean RNA concentration and A260/A280 ratio of all total RNA samples were 385 ± 64 µg/mL and 1.91 ± 0.02 (*N* = 36 or *n* = 12/group) and 466 ± 62 µg/mL and 1.93 ± 0.02 (*N* = 36 or *n* = 12/group) for liver and cecum mucosa, respectively. The synthesis of cDNA was performed as described previously (Chiappisi et al., 2017). qPCR analysis of target and reference genes was performed with a Rotor-Gene Q system (Qiagen) using the KAPA SYBR FAST qPCR Mastermix (Merck, Darmstadt, Germany) and gene-specific primer pairs from Eurofins MWG Operon (Ebersberg, Germany), as recently described (Meyer et al., 2019). The properties of primers are listed in Supplemental Table S1 for cecum mucosa. The qPCR data were normalized using 4 reference genes (*ACTB, GAPDH, SDHA* and *YWHAZ*) (Vandesompele et al., 2002). Also, qPCR analysis was carried out for validation of microarray data (17 genes) of the liver. The characteristics of these primers are listed in Supplemental Table S2.

### Determination of Triglyceride and Cholesterol Concentration in Plasma and Liver

The plasma and liver concentrations of triglycerides (**TG**) and cholesterol (**Chol**) were determined by using enzymatic reagent kits from DiaSys (Triglycerides FS LOT: 60161285; Cholesterol FS LOT: 50302128/34859, both from DiaSys Diagnostic Systems GmbH, Holzheim, Germany). The concentration of TG and Chol in the liver were measured by preparing liver lipid extracts using a mixture of n-hexane and isopropanol (3:2, v/v) according to Hara and Radin (1978). Afterwards, extracted lipids were dried and dissolved with chloroform and Triton X-100 (1:1, v/v). Chloroform was evaporated by a stream of nitrogen. The test reagents were added to the lipids dissolved in Triton X-100 (Eder and Kirchgessner, 1994).

### Hepatic Transcript Profiling

Following a further RNA quality check by an Agilent 2100 Bioanalyzer, which revealed an average RNA integrity number value of 7.49 ± 0.48 for all samples (*N* = 18, means ± SD), total RNA samples were processed using an Affymetrix GeneChip Array (Chicken Gene 1.0 ST), according to the Applied Biosystems GeneChip Whole Transcript (**WT**) PLUS Reagent Kit User Guide (Thermo Fisher Scientific, Waltham, MA). After scanning of the processed GeneChips, cell intensity files, where a single intensity value for each cell is provided, were generated from the image data using the Command Console software (Affymetrix). The compressed array image files (CEL files) were imported into the Applied Biosystems Transcriptome Analysis Console (v. 4.0.2) software (Thermo Fisher Scientific) for calculation of summarized probe set signals (in log2 scale) using the Robust Multichip Analysis algorithm, comparison fold changes (**FC**) and significance *P*-values (**ANOVA**). Gene names were annotated with the “ChiGene-1_0-st-v1.na36.galgal3.transcript.csv” annotation file. The microarray data of this study have been deposited in MIAME compliant format in the NCBI´s Gene Expression Omnibus (**GEO**) public repository under GEO accession number GSE269154 (Edgar et al., 2002). The differentially expressed transcripts were filtered based on a FC > 1.3 or < -1.3 and a *P*-value < 0.05 between groups PSA-5.0 vs. PSA-0 and groups PSA-2.5 vs. PSA-0. Identical or similar filter criteria were also applied in several recent studies (Ringseis et al., 2019; Grundmann et al., 2023; Schäfer et al., 2023). Gene set enrichment analysis (**GSEA**) was performed with the identified differentially expressed transcripts in order to identify enriched Gene Ontology (**GO**) terms within GO category biological process (**BP**), molecular function (**MF**) and cellular component (**CC**) and Kyoto Encyclopedia of Genes and Genomes (**KEGG**) pathways using the Database for Annotation, Visualization and Integrated Discovery bioinformatic resource 6.8 (Huang et al., 2009; Sherman et al., 2022). GO terms were considered as enriched if *P* < 0.05.

### Targeted Plasma Metabolomics

Targeted quantification of 1019 plasma metabolites was performed using a combination of liquid chromatography (Agilent 1290 infinity II LC, Santa Clara, CA) and mass spectrometry (SCIEX 5500 QTrap MS, Darmstadt, Germany) with the MxP Quant 500XL kit (BIOCRATES Life Sciences AG, Innsbruck, Austria) as described recently in detail (Marschall et al., 2023). Prior to the analysis, variables with missing values were either excluded from the analyses if > 50% of the samples were missing, or the missing values were replaced by the limit of detection (1/5 of the minimum positive value of each variable). Metabolites were filtered based on their interquartile range. After normalization by logarithmic transformation and autoscaling, the remaining values (507) were used for statistical analysis and principal component analysis (**PCA**).

### Statistical Analysis

Statistical analysis was performed using SPSS 28 software (IBM, Armonk, NY). The cage served as the experimental unit for feed intake and feed:gain ratio and the individual animal for all other data. All parameters were tested for normal distribution by Kolmogorov-Smirnov test for initial body weight, final body weight and body weight gain (all animals alive at the end of the trail, *N* = 65 broilers or *n* = 21-22 broilers/group) and the Shapiro-Wilk test for feed intake, feed:gain ratio (cage basis, *N* = 18 cages or *n* = 6 cages/group) and biochemical and qPCR data (2 animals per cage, *N* = 36 or *n* = 12 broilers/group). For test of homoscedasticity the Levene's test was used. If normal distribution was only observed after a log-transformation, the log-transformed data were used for statistical analysis. Differences between the 3 groups were analyzed using one-way analysis of variance (one-way **ANOVA**) followed by a Tukey's post-hoc test for normally distributed data with homogeneous variances. For data with heterogeneity of variance, means of the 3 groups were analyzed using Welch's ANOVA in conjunction with the Games-Howell post-hoc test. In the case that data were not-normally distributed, a Kruskal–Wallis one-way ANOVA was performed using the Mann-Whitney U test with Bonferroni correction as post-hoc test. For all the above-mentioned tests, a *P*-value < 0.05 was considered statistically significant. Statistical analysis of the metabolomics dataset was performed using MetaboAnalyst version 6.0 (Xia et al., 2009). Metabolites with a false discovery rate (**FDR**) < 0.05 were considered significantly different.

## RESULTS

### Chemical Composition of *P. sapidus* Mycelium

After 6 d of cultivation in a 150 L stirred bioreactor the *P. sapidus* mycelium contained (per kg DM) 105 g CP, 18 g EE, 36 g CA, and 780 g carbohydrates. The concentration of total glucans, β- and α-glucans (per kg DM) were 366 g, 312 g and 54 g, respectively. The chitin concentration of the mycelium was 53 g per kg DM. The concentrations of amino acids are shown in Supplemental Table S3. The highest concentrations within amino acids were detected for glutamine + glutamic acid and alanine. Concentrations of most of the other amino acids (arginine, asparagine + aspartic acid, cysteine, glycine, histidine, isoleucine, leucine, lysine, methionine, phenylalanine, proline, serine, threonine, tyrosine, and valine) were below 10 g/kg mycelium (on a DM base). The concentration of tryptophan was below a concentration of 1 g/kg mycelium (on a DM base). Fatty acid analysis showed that eight fatty acids were present with a concentration greater than 0.1% of total fatty acids in the mycelium. The highest concentration was found for C18:2, followed by C18:1, C16:0, C18:0 and C18:3. The concentrations of C12:0, C14:0 and C17:0 were below 1% of total fatty acids.

### Chemical Composition of the Experimental Diets

The concentration of nutrients of the 3 experimental diets fed during the 3 phases are shown in Table 2. Within one phase, the concentrations of crude nutrients, AME*_N_*, fatty acids and amino acids were similar within the 3 diets (PSA-0, PSA-2.5, PSA-5.0).

**Table 2:** Concentrations of nutrients and energy in the broiler diets supplemented with 0% (PSA-0), 2.5% (PSA-2.5) or 5% (PSA-5.0) of *Pleurotus sapidus* mycelium.

	Starter diets			Grower diets			Finisher diets		
	PSA-0	PSA-2.5	PSA-5.0	PSA-0	PSA-2.5	PSA-5.0	PSA-0	PSA-2.5	PSA-5.0
Analyzed crude nutrient and energy content									
DM (% FM)	86.1	86.4	86.6	86.6	86.5	86.9	86.8	86.5	86.9
CP (% DM)	24.4	24.3	24.4	22.9	22.9	22.9	21.1	21.2	21.2
EE (% DM)	9.1	8.9	8.9	8.5	8.5	8.3	8.8	9.1	8.7
CA (% DM)	6.5	6.6	6.6	6.1	6.2	6.3	6.3	6.4	6.5
CF (% DM)	5.8	5.5	5.1	5.2	4.7	4.8	5.1	4.1	3.9
Sugar (% DM)	3.8	2.8	5.0	3.0	4.8	5.4	3.2	3.7	5.3
Starch (% DM)	38.3	38.4	35.5	41.2	39.1	40.1	44.9	44.0	44.5
Calculated energy content									
AME*_N_* (MJ/kg DM)	13.8	13.6	13.4	13.7	13.6	13.8	14.2	14.2	14.4
Fatty acids (% of total fatty acids)^1^									
C16:0	11.54	11.56	11.60	11.48	11.37	11.64	11.57	11.47	11.56
C18:0	3.63	3.86	4.25	3.55	3.94	4.31	4.04	3.96	4.11
C18:1	26.39	25.19	23.79	26.53	24.15	23.75	23.71	23.80	23.79
C18:2	52.34	53.21	53.67	52.56	54.05	53.88	54.28	54.33	54.11
C18:3	4.62	5.02	5.36	4.58	5.33	5.35	5.27	5.31	5.32
C20:0	0.40	0.37	0.42	0.38	0.35	0.37	0.35	0.35	0.36
C20:1	0.31	0.25	0.31	0.33	0.29	0.21	0.31	0.30	0.28
C22:0	0.43	0.39	0.34	0.43	0.34	0.33	0.31	0.31	0.32
Amino acids (g/kg diet)									
Alanine	8.96	9.05	9.19	8.31	8.33	8.81	7.80	8.07	8.27
Arginine	15.46	14.21	14.68	13.68	13.33	13.51	12.15	12.50	12.45
Asparagine/Aspartic acid	18.93	17.58	17.82	16.09	15.91	16.52	14.66	15.08	14.94
Cysteine	3.19	3.30	3.04	3.02	2.85	2.97	2.76	3.12	3.14
Glutamine/Glutamic acid	36.93	35.97	36.11	35.57	34.59	35.40	33.14	32.98	32.69
Glycine	7.95	8.01	8.09	7.56	7.40	7.60	6.89	6.97	7.00
Histidine	5.14	4.78	4.87	4.51	4.52	4.45	4.20	4.26	4.25
Isoleucine	8.77	8.63	8.90	8.83	8.67	8.92	8.06	8.14	7.97
Leucine	15.77	15.20	15.29	14.50	14.47	14.56	13.76	13.72	13.62
Lysine	14.84	13.63	13.78	13.09	12.96	12.96	12.01	12.20	11.89
Methionine	6.39	6.37	6.29	6.21	6.39	6.21	5.61	6.03	5.70
Phenylalanine	10.34	9.41	9.59	9.08	9.00	9.04	8.40	8.31	8.43
Proline	13.62	13.61	13.48	14.16	13.54	13.21	13.07	12.78	12.73
Serine	10.25	9.96	10.11	9.63	9.31	9.62	8.75	8.87	8.86
Threonine	9.11	9.29	9.43	8.88	8.50	8.81	7.85	8.13	7.98
Tryptophan	3.47	3.42	3.12	2.82	2.86	2.76	2.82	2.91	2.96
Tyrosine	6.59	6.52	6.37	5.72	5.77	5.21	5.39	5.47	4.85
Valine	10.14	9.61	10.13	10.28	10.01	10.08	9.01	9.03	8.92

^1^Only fatty acids > 0.1% of total fatty acids are shown.

Abbreviations: CA, crude ash; CF, crude fiber; CP, crude protein; DM, dry matter; EE, ether extract; FM, fresh matter.

### Performance and AID of Nutrients

Final body weight (*P* = 0.881), body weight gain (*P* = 0.866), feed intake (*P* = 0.515), and feed:gain ratio (*P =* 0.846) of the broilers during the whole period did not differ between the 3 groups of broilers (Table 3). The mortality of the broilers was 8.3% in group PSA-0 and 4.2% in groups PSA-2.5 and PSA-5.0. The AID of CP (*P* = 0.769), EE (*P* = 0.316) and amino acids (lowest *P*-value: 0.347 for cysteine, highest *P*-value: 0.997 for tryptophan) did also not differ between the 3 groups (Table 4).

**Table 3:** Performance data of broilers fed diets with either 0% (PSA-0), 2.5% (PSA-2.5) or 5.0% (PSA-5.0) *Pleurotus sapidus* mycelium for 35 d.

	PSA-0	PSA-2.5	PSA-5.0	*P*-value
Whole period (d 1–35)				
Initial BW (g)	38.1 ± 3.0	38.0 ± 2.9	38.1 ± 2.9	0.995
Final BW (g)	2870 ± 328	2940 ± 243	2911 ± 300	0.881
BW gain (g)	2832 ± 328	2902 ± 242	2873 ± 300	0.866
Feed intake (g)	3827 ± 238	3960 ± 154	3885 ± 245	0.515
Feed:gain ratio (g/g)	1.35 ± 0.08	1.37 ± 0.06	1.36 ± 0.03	0.846
Mortality (%)	8.3	4.2	4.2	-
Starter period (d 1–10)				
BW gain (g)	245 ± 35	261 ± 29	260 ± 27	0.171
Feed intake (g)	254 ± 27	282 ± 15	273 ± 23	0.165
Feed:gain ratio (g/g)	1.03 ± 0.08	1.08 ± 0.05	1.05 ± 0.04	0.203
Grower period (d 11–21)				
BW gain (g)	821 ± 77	855 ± 78	841 ± 81	0.361
Feed intake (g)	1001 ± 117	1146 ± 105	1072 ± 40	0.062
Feed:gain ratio (g/g)	1.22 ± 0.13	1.35 ± 0.15	1.27 ± 0.02	0.402
Finisher period (d 22–35)				
BW gain (g)	1761 ± 285	1784 ± 204	1760 ± 260	0.820
Feed intake (g)	2572 ± 193	2531 ± 131	2540 ± 230	0.979
Feed:gain ratio (g/g)	1.47 ± 0.10	1.43 ± 0.04	1.46 ± 0.05	0.556

Data are means ± SD, *n* = 21-22 broilers/group (initial BW, final BW, BW gain) and *n* = 6 cages/group (feed intake, feed:gain ratio).

Abbreviation: BW, body weight.

**Table 4:** Apparent ileal digestibility (AID) of ether extract (EE), crude protein (CP) and amino acids of broilers fed diets with either 0% (PSA-0), 2.5% (PSA-2.5) or 5.0% (PSA-5.0) *Pleurotus sapidus* mycelium for 35 d.

AID	PSA-0	PSA-2.5	PSA-5.0	*P*-value
EE (%)	89.7 ± 1.5	89.7 ± 1.9	88.5 ± 2.2	0.316
CP (%)	76.1 ± 2.6	75.2 ± 4.5	74.7 ± 5.1	0.769
Amino acids (%)				
Alanine	74.5 ± 4.5	73.2 ± 7.3	74.3 ± 5.6	0.845
Arginine	88.7 ± 1.9	87.9 ± 33	86.6 ± 4.7	0.394
Asparagine/Aspartic acid	80.7 ± 2.3	80.6 ± 3.4	80.5 ± 4.0	0.983
Cysteine	70.5 ± 3.9	73.7 ± 4.1	72.5 ± 6.5	0.347
Glutamine/Glutamic acid	87.4 ± 2.3	86.6 ± 3.7	86.2 ± 4.7	0.746
Glycine	76.6 ± 3.2	75.8 ± 5.3	76.5 ± 5.6	0.923
Histidine	83.5 ± 2.8	82.0 ± 5.1	82.5 ± 5.4	0.742
Isoleucine	84.1 ± 2.3	83.3 ± 4.5	83.2 ± 5.0	0.860
Leucine	83.2 ±.2.9	82.0 ± 4.6	81.9 ± 5.2	0.783
Lysine	87.9 ± 2.1	87.1 ± 3.9	86.8 ± 4.2	0.731
Methionine	89.3 ± 2.0	89.5 ± 3.2	88.8 ± 4.2	0.863
Phenylalanine	84.0 ± 2.9	82.7 ± 4.8	82.6 ± 5.6	0.732
Proline	87.0 ± 3.1	86.3 ± 4.0	86.6 ± 4.2	0.830
Serine	79.9 ± 3.2	79.4 ± 5.2	79.6 ± 5.8	0.972
Threonine	79.2 ± 3.4	78.8 ± 6.0	79.4 ± 5.8	0.862
Tryptophan	82.9 ± 2.5	83.1 ± 3.2	81.9 ± 7.0	0.997
Tyrosine	82.3 ± 2.8	80.9 ± 5.8	81.3 ± 5.9	0.828
Valine	84.3 ± 2.6	83.2 ± 5.0	83.7 ± 4.8	0.850

Data are means ± SD, *n* = 12 broilers/group.

### Diversity and Composition of the Cecum Microbiota

**Figure 1:**
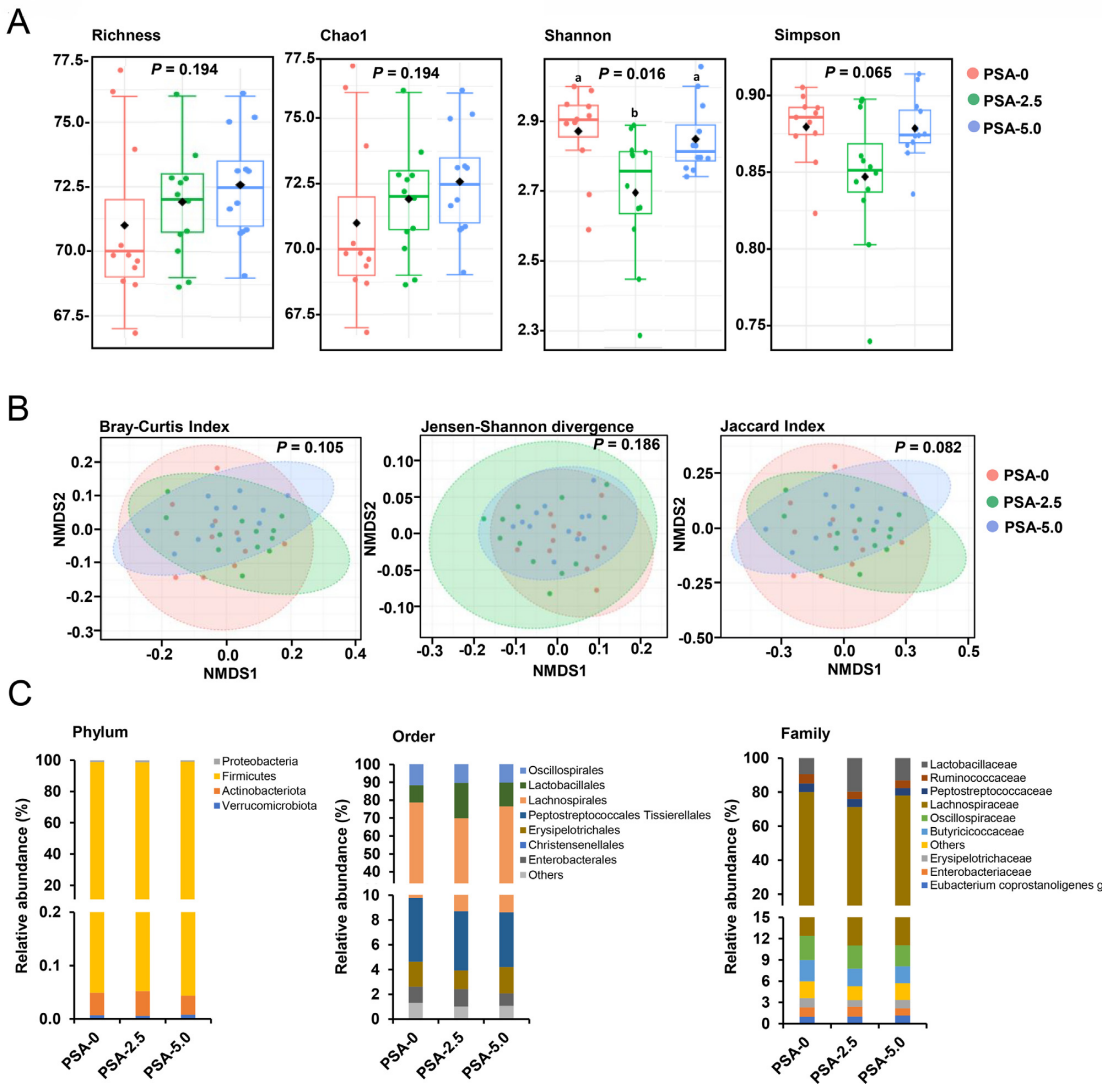
Analysis of the cecal microbiota. Indicators of α-diversity (Richness, Chao1, Shannon Index and Simpson Index) of the cecal bacterial community **(A),** visualization of the difference in the β-diversity (Bray-Curtis Index, Jenson-Shannon divergence and Jaccard Index) of cecal community between the groups by NMDS plots **(B)** and taxonomic composition (phylum, order, family) **(C)** of the cecal bacteria of broilers fed diets with either 0% (PSA-0), 2.5% (PSA-2.5) or 5% (PSA-5.0) *P. sapidus* mycelium for 35 d. (**A)** box plot for *n* = 12 broilers/group; (**B)** NMDS plots for *n* = 12 broilers/group; (**C)** Data are means for *n* = 12 broilers/group.

After normalizing the data and a subsequent filtering step, a total of 80 ASV was used to analyze the diversity and composition of the microbiota. The 4 indicators Richness, Chao1, Shannon-Index and Simpson-Index were used to analyze the α-diversity (Figure 1A). Richness and Chao1, which are both indicators of the microbial community richness, showed no significant differences between the groups. The Shannon-Index, which considers both the richness and the evenness, differed significantly (*P* < 0.05) between group PSA-0 and group PSA-2.5, but not between group PSA-0 and PSA-5.0, while no effect was found for the Simpson-Index. To assess β-diversity, 3 metrics (Bray-Curtis Index, Jensen-Shannon divergence, Jaccard Index) were calculated and the data were visualized using nonmetric multidimensional scaling (**NMDS**) plots (Figure 1B). The β-diversity metrics indicated no significant difference between the groups. The effects on the composition of the microbiota were analyzed at different taxonomic levels (Figure 1C). The analysis at the phylum level showed that the cecal digesta samples contained bacteria from only 4 different phyla, whose abundance did not differ between the 3 groups. The predominant phylum was Firmicutes with > 98% in all groups, followed by Proteobacteria (1.01–1.40% in all groups), Actinobacteriota (0.03–0.04% in all groups) and Verrumicrobiota (< 0.01% in all groups). At the order level, 14 orders were identified with Lachnospirales (60–67% in all groups), Oscillospirales (11–13%) and Lactobacillaes (9-19%) as dominant taxa. However, only 2 low-abundance bacterial taxa (Clostridia UCG 014 and Eubacteriales, both belonging to the phylum Firmicutes) differed across the groups (*P* < 0.05). Clostridia UCG 014 showed higher abundance in the group PSA-0 and group PSA-5.0 than in group PSA-2.5, while the abundance of Eubacteriales was higher in group PSA-5.0 than in the other 2 groups and higher in group PSA-2.5 than in group PSA-0. At the family level, 23 different families were identified and only 1 low-abundance bacterial family (Anaerofustaceae), belonging to the class Clostridia and phylum Firmicutes, was affected by treatment (*P* < 0.05). The abundance of Anaerofustaceae was higher in group PSA-5.0 compared to the other 2 groups and higher in group PSA-2.5 compared to group PSA-0. The abundance of all bacterial taxa in the cecal digesta of the broilers is shown in Supplemental Table S4.

### Cecal Digesta Concentrations of SCFA

**Figure 2:**
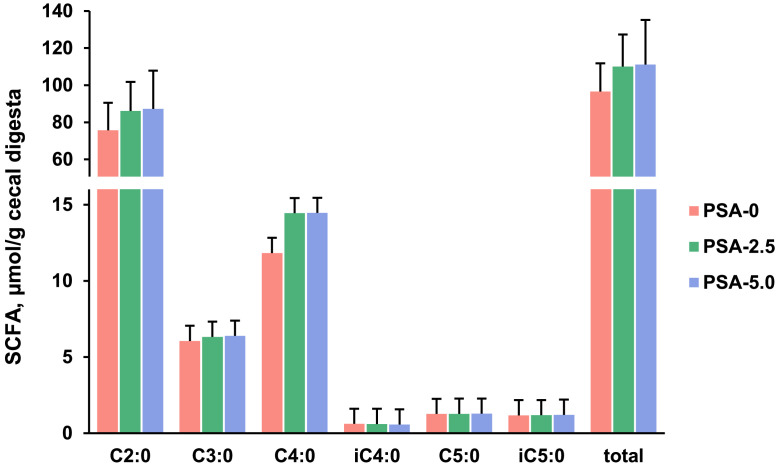
Concentrations of individual (acetic acid (C2:0), *P* = 0.227; propionic acid (C3:0), *P* = 0.739; butyric acid (C4:0), *P* = 0.253; isobutyric acid (iC4:0), *P* = 0.896; valeric acid (C5:0), *P* = 0.983; isovaleric acid (iC5:0), *P* = 0.958) and total (= sum of all individual, *P* = 0.157) short-chain fatty acids (**SCFA**) in cecal digesta of broilers fed diets with either 0% (PSA-0), 2.5% (PSA-2.5) or 5% (PSA-5.0) *P. sapidus* mycelium for 35 d. Data are means ± SD, *n* = 12 broilers/group.

The concentrations of total and individual SCFA (C2:0, acetic acid; C3:0, propionic acid; C4:0, butyric acid; iC4:0, isobutyric acid; C5:0, valeric acid; iC5:0, isovaleric acid) in the cecal digesta were not different between the 3 groups of broilers (Figure 2).

### Concentration of LPS in Plasma and mRNA Levels of Genes Involved in Gut Integrity in Cecum Mucosa

The concentration of LPS in plasma did not differ between the 3 groups of broilers. The mRNA levels of genes encoding tight-junction proteins (*CLDN3, CLDN5, JAM2* and *OCLN*), mucins (*MUC2, MUC5AC* and *MUC13*) and pro-inflammatory genes (*IL1b, IL8L1, IL8L2, TLR4* and *VCAM1*) in cecum mucosa were not different between the 3 groups (Table 5).

**Table 5:** Concentrations of lipopolysaccharides in plasma and mRNA levels of genes in cecum mucosa of broilers fed diets with either 0% (PSA-0), 2.5% (PSA-2.5) or 5.0% (PSA-5.0) *Pleurotus sapidus* mycelium for 35 d.

	PSA-0	PSA-2.5	PSA-5.0	*P*-value
Lipopolysaccharides (pg/ml)	22.9 ± 20.4	17.0 ± 12.0	8.21 ± 6.01	0.155
Normalized mRNA level (fold of PSA-0)				
Tight-junction proteins				
*CLDN3*	1.00 ± 0.69	0.92 ± 0.70	0.76 ± 0.31	0.747
*CLDN5*	1.00 ± 0.55	1.36 ± 1.09	1.17 ± 0.69	0.848
*JAM2*	1.00 ± 0.86	0.93 ± 0.66	1.1 ± 0.94	0.973
*OCLN*	1.00 ± 0.42	0.96 ± 0.56	1.17 ± 0.44	0.558
Mucins				
*MUC2*	1.00 ± 0.72	1.52 ± 0.97	1.22 ± 0.73	0.384
*MUC5AC*	1.00 ± 0.57	0.88 ± 0.25	0.71 ± 0.44	0.531
*MUC13*	1.00 ± 0.50	1.25 ± 0.62	1.13 ± 0.55	0.590
Proinflammatory genes				
*IL1B*	1.00 ± 0.69	1.24 ± 0.40	1.07 ± 0.52	0.268
*IL8L1*	1.00 ± 0.58	0.93 ± 0.56	0.96 ± 0.54	0.831
*IL8L2*	1.00 ± 0.57	1.02 ± 0.57	0.99 ± 0.42	0.970
*TLR4*	1.00 ± 0.77	1.12 ± 0.59	0.83 ± 0.43	0.568
*VCAM1*	1.00 ± 0.95	1.22 ± 0.73	1.16 ± 0.55	0.344

Data are means ± SD, *n* = 12 broilers/group.

^a,b^Means without a common letter differ across the groups, *P* < 0.05.

### Liver Weight and Concentrations of TG and Chol in Liver and Plasma

Liver weights and concentrations of TG and Chol in liver and plasma are shown in Table 6. The liver weight was significantly higher in broilers of group PSA-5.0 than in broilers of group PSA-0 (*P* < 0.05), but did not differ between group PSA-5.0 and group PSA-2.5 and between group PSA-2.5 and group PSA-0. The relative liver weight did not differ between the 3 groups. While the concentrations of TG and Chol in plasma and concentration of Chol in liver did not differ between the groups, the concentration of TG in liver was higher in broilers of group PSA-5.0 than in broilers of group PSA-2.5 (*P* < 0.05). The concentration of TG in the liver did not differ between broilers of group PSA-0 and broilers of group PSA-5.0.

**Table 6:** Liver weight and triglycerides and cholesterol in liver and plasma of broilers fed diets with either 0% (PSA-0), 2.5% (PSA-2.5) or 5.0% (PSA-5.0) *Pleurotus sapidus* mycelium for 35 d.

	PSA-0	PSA-2.5	PSA-5.0	*P*-value
Liver weight (g)	49.9 ± 4.8^b^	54.0 ± 6.8^ab^	57.3 ± 4.6^a^	0.044
Liver weight (% of BW)	1.73 ± 0.16	1.83 ± 0.23	1.92 ± 0.14	0.059
Liver				
Triglycerides (µmol/g)	13.6 ± 2.8^ab^	13.0 ± 2.3^b^	16.7 ± 4.0^a^	0.021
Cholesterol (µmol/g)	12.4 ± 0.8	11.7 ± 1.9	13.3 ± 2.0	0.158
Plasma				
Triglycerides (mmol/L)	0.57 ± 0.26	0.53 ± 0.22	0.74 ± 0.27	0.121
Cholesterol (mmol/L)	2.90 ± 0.71	2.88 ± 0.47	3.10 ± 0.45	0.276

Data are means ± SD, *n* = 12 broilers/group.

^a,b^Means without a common letter differ across the groups, *P* < 0.05. Abbreviation: BW, body weight.

### Liver Transcriptome

**Figure 3:**
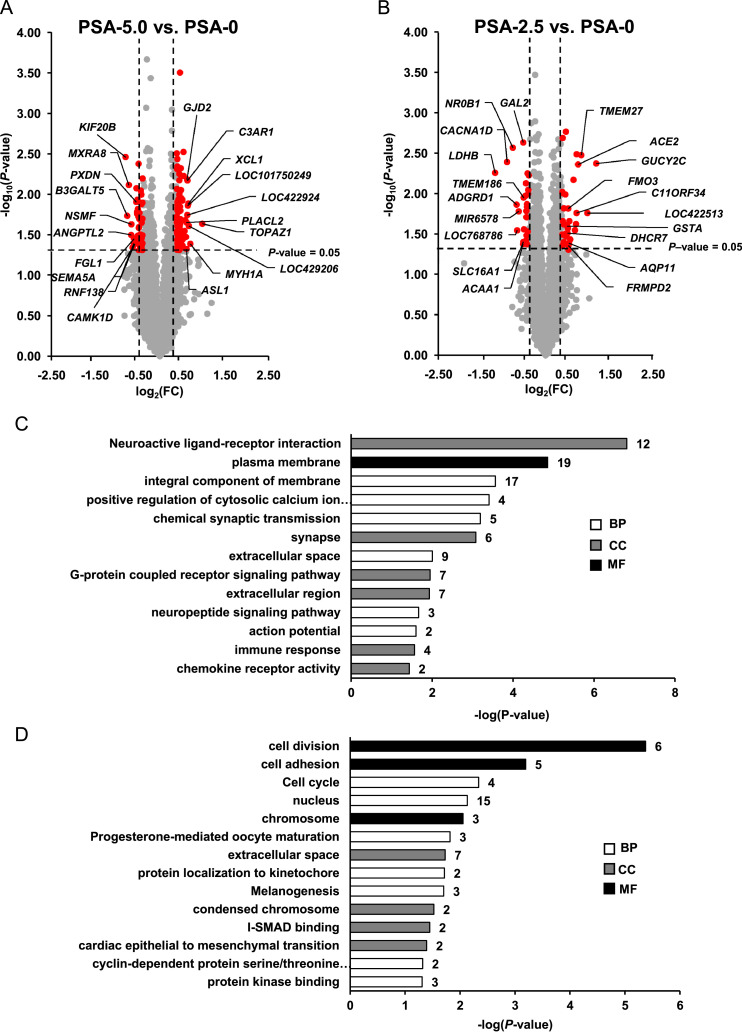
Differential transcriptome analysis in the liver (*n* = 6 microarrays/group). Volcano plot illustrating the differentially expressed transcripts in the liver of broilers between group PSA-5.0 vs. PSA-0 **(A)** and group PSA-2.5 vs. PSA-0 **(B)**. The filtering criteria are indicated by horizontal (*P*-value = 0.05) and vertical (fold change >1.3 or < -1.3) dashed lines. Red dots in the upper left and upper right corner represent the downregulated and upregulated transcripts. Most enriched gene ontology (GO) terms (biological process (BP), cellular component (CC) and molecular function (MF)) associated with the differentially expressed upregulated **(C)** and downregulated **(D)** transcripts between group PSA-5.0 vs. PSA-0. GO terms are sorted by their enrichment *P*-values (EASE score) (top: lowest *P*-value, bottom: highest *P*-value).

According to the filter criteria applied (*P* < 0.05; FC >1.3 and < - 1.3), a total of 144 transcripts were identified as differentially expressed (upregulated: 94, downregulated: 50) in the liver between group PSA-5.0 and group PSA-0. The volcano plot (Figure 3A) illustrates the differentially expressed transcripts between group PSA-5.0 and group PSA-0 as red dots. The top 10 upregulated transcripts in decreasing order of their FC (in brackets) were: *TOPAZ1* (1.97), *MYH1A* (1.63), *LOC429206* (1.59), *XCL1* (1.58), *LOC101750249* (1.56), *C3AR1* (1.55), *LOC422924* (1.55), *GJD2* (1.55), *ASL1* (1.53) and *PLACL2* (1.53). The top 10 downregulated transcripts in increasing order of their FC (in brackets) were: *KIF20B* (-1.73), *B3GALT5* (-1.68), *MXRA8* (-1.64), *ANGPTL2* (-1.58), *NSMF* (-1.58), *CAMK1D* (-1.54), *RNF138* (-1.51), *FGL1* (-1.51), *SEMA5A* (-1.49) and *PXDN* (-1.46). The FC and *P*-value of all differentially expressed transcripts between group PSA-5.0 vs. PSA-0 are shown in Supplemental Table S5.

Considering the same filter criteria as for the comparison of group PSA-2.5 and group PSA-0, a total of 58 transcripts were identified as differentially expressed (upregulated: 34, downregulated: 24) in the liver (Figure 3B). Only one gene (*GUCY2C*) was regulated > 2.0-fold and one gene (*LDHB*) < -2.0-fold. The 10 most strongly upregulated transcripts in decreasing order of their FC (in brackets) were: *GUCY2C* (2.27), *LOC422513* (1.97), *TMEM27* (1.78), *ACE2* (1.69), *C11ORF34* (1.66), *LOC100859846* (1.65), *DNAJC6* (1.64), *CYP2K1L* (1.61), *OVSTL* (1.57) and *LOC428289* (1.49). The 10 most strongly downregulated transcripts in increasing order of their FC (in brackets) were: *LDHB* (-2.26), *CACNA1D* (-1.87), *NR0B1* (-1.70), *ADGRD1* (-1.60), *LOC768786* (-1.59), *MIR6578* (-1.54), *ACAA1* (-1.44), *SLC16A1* (-1.44), *GAL2* (-1.44) and *TMEM186* (-1.43). The FC and *P*-value of all differentially expressed transcripts between group PSA-2.5 and group PSA-0 are shown in Supplemental Table S6.

Microarray data of 17 differentially expressed transcripts between groups PSA-5.0 and PSA-0 were validated by qPCR. As shown in Supplemental Table S7, the effect direction (positive or negative FC) was the same between microarray and qPCR for all validated transcripts, whereas the effect size (value of FC) differed to some extent for the validated transcripts between microarray and qPCR. Statistical analysis of qPCR data revealed that only 2 transcripts were regulated significantly (*P*-value < 0.05) (*FYB1, PSTPIP2*), whereas 3 transcripts showed a *P*-value < 0.1 (*MYH1A, TOPAZ1, XCL1*). The other twelve transcripts were not regulated (*ANGPTL2, C3AR1L, CCNB1, HTR7, KITLG, PHC1, PLEK, SMC2, TBXAS1, TOP2A, TSHZ2, WWTR1*).

In order to extract biological meaning from the transcripts differentially expressed between groups PSA-5.0 and PSA-0, GSEA was performed using GO terms and KEGG-pathways. Due to the low number of differentially expressed transcripts, GSEA was not conducted for the comparison of groups PSA-2.5 and PSA-0. The GSEA identified 13 enriched terms within the upregulated genes. “Neuroactive ligand-receptor interaction” was the most strongly enriched GO term belonging to GO category cellular component (Figure 3C). The most strongly enriched KEGG-pathway was “integral component of plasma membrane pathway” (*P* = 0.041). Fourteen terms were identified to be enriched within the downregulated transcripts. “Cell division” was the most strongly enriched GO term (Figure 3D). Within the downregulated transcripts, “cell cycle”, “progesterone-mediated oocyte maturation” and “melanogenesis” were identified as enriched KEGG-pathways.

### Plasma Metabolome

**Figure 4:**
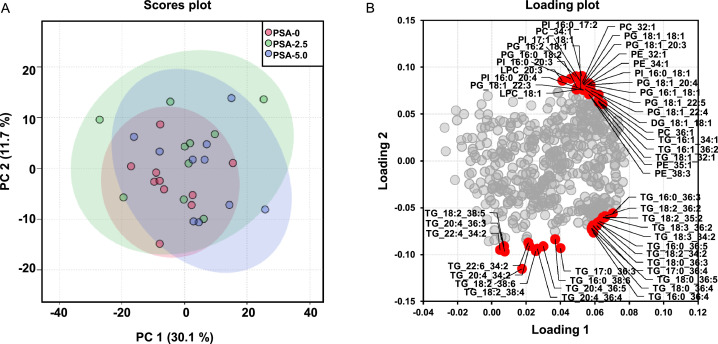
Principal component analysis (**PCA**) of plasma metabolome. Scores plot with plotted 5% confidence interval **(A)** and associated loading plot **(B)** of PCA of broilers plasma metabolome. Broilers were fed diets with either 0% (PSA-0), 2.5% (PSA-2.5) or 5.0% (PSA-5.0) *P.* sapidus mycelium for 35 d. Data are principal components (PC 1 or PC 2) and their loadings. The upper 10 % of the parameters with the greatest distance to zero are marked in red, *n* = 10 broilers/group. Abbreviations: DG, Diacylglycerols; LPC, lysophosphatidylcholine; PC, phosphatidylcholine; PE, phosphatidylethanolamine; PI, Phosphatidylinositols; TG, triglycerides.

The detected metabolites belonged to the group of TG (188), followed by phosphatidylethanolamines (59), phosphatidylcholines (49), phosphatidylinositols (38), amino acids (38), phosphatidylglycerols (26), Chol esters (18), phosphatic acids (17), diacylglycerols (11), fatty acids (11), ceramides (8), sphingomyelins (6), biogenic amines (5), carboxylic acids (4), monoacylglycerols (4), bile acids (3), glucosylceramides (3), sphingobases (3), indoles (2), nucleobases related (2), cresols (1), phosphatidylserines (1), sugars (1), and vitamins and cofactors (1). The concentration of all the analyzed metabolites did not differ between the 3 groups of broilers according to the FDR < 0.05. The score plot of the PCA also showed no separate clustering of the 3 groups (Figure 4). All analyzed metabolites are shown in Supplemental Table S8.

## DISCUSSION

Several studies have already reported beneficial effects (e.g., hypolipidemic, antioxidant, antibacterial, immune system-enhancing) of fungal fruiting bodies for humans and animals (Mattila et al., 2001; Hu et al., 2006; Valverde et al., 2015). However, knowledge about the effect of fungal mycelia produced by submerged cultivation as a part of feed for poultry is limited. Therefore, the present study was conducted to investigate the effects of a biotechnologically produced *P. sapidus* mycelium on performance, digestibility of nutrients, gut microbiota structure and intermediary metabolism in broilers.

Chemical analysis of the produced *P. sapidus* mycelium showed that carbohydrates were the dominating nutrient fraction making up nearly 80% of the mycelial DM. Similar contents of carbohydrates have been reported for other *P.* mycelia, for example, *P. eous* (former *P. sajor-caju*), produced by submerged cultivation (Maheshwari et al., 2020). Within the carbohydrate fraction, the β-glucans were the dominant compound reaching similar levels as reported for *P. eous* (Manzi, 2000; Maheshwari et al., 2020). Regarding that certain β-glucans are well-known to act as potent prebiotics in monogastric animals (Lam and Chi-Keung Cheung, 2013), the *P. sapidus* mycelium used in the present study could be considered as a rich source of prebiotics. The dietary inclusion level of 5% of the *P. sapidus* mycelium was chosen based on findings of a recent study in which the same dose of *P. eous* mycelium in the diet exhibited beneficial effects on hepatic lipid metabolism and gut function in an obese rat model (Maheshwari et al., 2020). The *P. sapidus* mycelium was also included in a lower dose (2.5%) in order to evaluate if the effects observed are dose-dependent. The fungal mycelium was included into the diets at the expense of wheat in order to keep the energy content of the 3 diets comparable. Isoenergetic inclusion of the fungal mycelium would have been also possible by replacing maize, but we are not aware of any reason that study results would have been substantially different when the mycelium was replaced at the expense of maize.

In the present study, dietary inclusion of 2.5% and 5% *P. sapidus* mycelium did not exert any effects on growth performance, feed intake, feed conversion and AID of EE, CP and amino acids of the broilers. Despite improvements of performance and nutrient digestibility have been documented in several studies with broilers fed β-glucans or other prebiotic oligosaccharides (Chae et al., 2006; Richards et al. 2020; Al-Baadani et al., 2022), there are also studies demonstrating no effect of β-glucans on broiler performance (Cox et al., 2010; Moon et al., 2016). These contradictory results have been attributed to the fact that the structures of different β-glucans can vary significantly which strongly affects physicochemical properties including solubility, viscosity, gelation, binding ability, bulking ability, and fermentability (Singh and Kim, 2021). Considering that improvements of broiler performance in response to feeding of prebiotic-enriched diets in the abovementioned studies were associated with positive effects on gut integrity and/or gut microbiota composition (Richards et al. 2020; Al-Baadani et al., 2022), we also studied the effect of dietary inclusion of the *P. sapidus* mycelium and, thus, a high content of β-glucans on the bacterial community structure and diversity in the gut of the broilers.

Evaluation of the effect of dietary inclusion of *P. sapidus* mycelium on the gut microbiota composition and diversity of the broilers based on metagenomic analysis of the cecum digesta revealed only marginal effects. Amongst the α-diversity metrics investigated, only 1 (Shannon-Index) out of 4 was found to be slightly affected, while all β-diversity indicators did not differ among groups. Likewise, analysis of the microbiota composition of the broilers at different taxonomic levels showed almost no effect of dietary treatment. The only exception was that the abundance of 2 low-abundance bacterial taxa belonging to the phylum Firmicutes (Clostridia UCG 014, Eubacteriales) was altered in the groups fed the *P. sapidus* mycelium. Thus, the lack of effect of the *P. sapidus* mycelium on broiler performance in the present study could be explained by its very weak effect on the gut microbiota structure, which might be caused by a limited fermentability of the β-glucans or other carbohydrates of the fungal mycelium. The latter assumption is supported by the observation that the concentrations of total and all individual SCFA in the cecal digesta of the broilers did not differ among groups. It can therefore be concluded that, in contrast to our assumption, the high β-glucan content of the *P. sapidus* mycelium had only marginal effects on the structure of the cecum microbiota community in healthy broilers. Despite these marginal effects, the lack of any untoward changes of the gut microbiota structure, like increases in the abundance of obligate pathogens, suggests that the *P. sapidus* mycelium can be fed to broilers without impairment of the gut microbial community.

In contrast to these weak effects of the *P. sapidus* mycelium on the gut microbiota in broilers, feeding a *P. eous* mycelium-enriched diet to obese rats caused a strong effect on the gut microbiota structure with increases in the abundance of beneficial bacterial taxa, such as *Lactobacillaceae, Bifidobacterium, Roseburia*, and *Blautia*, and a decrease in the abundance of the pathogenic genus *Escherichia−Shigella* (Maheshwari et al., 2021). In addition, the favorable effect of dietary *P. eous* mycelium on the gut microbiota in the obese rat model was associated with an improved gut barrier function as evidenced from decreased levels of the bacterial endotoxin LPS (Maheshwari et al., 2021). It is well-known that obese animals develop gut dysbiosis – a term used to describe a perturbation of the commensal gut bacteria. Gut dysbiosis causes an impairment of the gut barrier function due to gut mucosa inflammation, decreased production of mucins leading to thinning of the protective mucin layer and reduced production of tight-junction proteins, which tightly connect adjacent intestinal epithelial cells in order to prevent paracellular passage of bacteria from the gut lumen into the portal vein (Ringseis et al., 2020). Consequently, gut dysbiosis is associated with hyperpermeability of the gut and induction of endotoxemia and metabolic derangements (Ringseis et al., 2020).

In the present broiler study, no evidence was gained that gut integrity was affected by dietary inclusion of the *P. sapidus* mycelium. This assumption is based on the observation that plasma levels of LPS and the mRNA levels of proinflammatory genes (*IL1B, IL8L1, IL8L2, TLR4, VCAM1*), tight-junction proteins (*CLDN3, CLDN5, JAM2, OCLN*), and mucins (*MUC2, MUC5AC, MUC13*) in the cecum mucosa did not differ among groups. These findings indicated that the key protective elements of the gut barrier were not impaired and no substantial translocation of bacterial compounds occurred in response to dietary inclusion of the *P. sapidus* mycelium in the broilers. This effect of feeding the *P. sapidus* mycelium can be considered as favorable considering that no impairment of gut integrity is beneficial and an improvement of gut integrity in healthy broilers with a normal gut microbiota structure is unlikely. In contrast to healthy broilers, feeding a *P. eous* mycelium to obese rats suffering from gut dysbiosis is able to restructure the perturbed gut microbiota, thereby, improving gut integrity.

Apart from marginal or absent effects of feeding the β-glucan-rich *P. sapidus* mycelium on the gut microbiota and gut integrity, our study revealed only very few effects on the intermediary metabolism of the broilers. The gut microbiota is well-known to strongly affect animal´s intermediary metabolism and feeding behavior *via* the gut-liver-brain axis, and gut-derived compounds, such as SCFA or bile acids, serve as the communication signals between gut microbes and all key metabolic tissues (Ringseis et al., 2020). In order to evaluate if the intermediary metabolism of the broilers was affected by feeding the *P. sapidus* mycelium, we carried out genome-wide hepatic transcript profiling. According to this analysis, the *P. sapidus* mycelium had only a very moderate effect on the hepatic transcriptome, which was particularly evident from the very weak regulation of the genes identified to be differentially expressed between group PSA-5.0 and PSA-0. None of these genes was regulated greater 2-fold and *TOPAZ1* (1.97-fold) and *KIF20B* (-1.73-fold) were the top up- and down-regulated genes, respectively. Considering the above-mentioned connection between gut microbiota and host metabolism, the weak effect of the *P. sapidus* mycelium on the hepatic transcriptome is in agreement with the very weak effect on the cecal microbiota and the lack of effect on cecal SCFA concentrations. Despite the very weak effect on the hepatic transcriptome, liver weights and liver TG concentration but not liver Chol concentration were slightly higher in the broilers of group PSA-5.0 compared to group PSA-0. Since hepatic transcript profiling did not reveal an up-regulation of hepatic genes involved in lipid synthesis, such as *ACACA, FASN, SCD, HMGCR*, and genes involved in inflammation and stress-response, such as *VCAM1, TLR4, IL8L1*, in group PSA-5.0 compared to group PSA-0, the mechanism underlying the increase of hepatic TG concentration is unknown. However, considering that the hepatic TG concentration in broilers of group PSA-5.0 was still within the physiological range, this slight increase should not be overstated. Based on this, the effect of the *P. sapidus* mycelium on hepatic metabolism can be regarded as very moderate. Our assessment of a weak effect of the *P. sapidus* mycelium on the intermediary metabolism of broilers is supported by the results from targeted metabolomics of blood plasma and from the measurement of plasma TG and Chol concentrations. According to plasma metabolomics, the concentrations of none of the large set of metabolites analyzed differed across the groups. This was also reflected by PCA showing no separation of the metabolomes of the different groups. Despite our study revealed no obvious improvements of intermediary metabolism of the broilers due to dietary inclusion of the *P. sapidus* mycelium, the lack of any adverse metabolic effects, which is a prerequisite for the use of novel feed components in animal nutrition, can be considered as beneficial.

## CONCLUSIONS

The present study shows that dietary inclusion of 2.5% and 5% of a biotechnologically produced *P. sapidus* mycelium in broiler diets does not affect growth performance and nutrient digestibility. In addition, feeding of the *P. sapidus* mycelium exhibited only a negligible effect on the cecal microbiota community but did not affect the concentrations of microbial fermentation products and did not impair gut barrier function. In line with this, liver transcriptomic and plasma metabolomics revealed only weak or not any effects, respectively, of feeding the *P. sapidus* mycelium in the broilers. Based on these findings it can be concluded that inclusion of a sustainably produced fungal mycelium in broiler diets at the expense of other feed components, such as wheat, has no negative consequences on broilers´ performance and metabolism. However, it cannot be excluded that higher doses of *P. sapidus* mycelium achieve different results.

## DISCLOSURES

The authors declare no conflicts of interest.

## Appendix Supplementary materials

Image, application 1

## Footnotes

Supplementary material associated with this article can be found in the online version at doi:10.1016/j.psj.2024.103975.
