# Supplementary material for: Effects of a biotechnologically produced Pleurotus sapidus mycelium on gut microbiome, liver transcriptome and plasma metabolome of broilers
Source: Poult Sci. 2024 Jun 13;103(9):103975. doi: 10.1016/j.psj.2024.103975 (PMC11261454; doi:10.1016/j.psj.2024.103975)
Supplement: Supplementary file 1 [file mmc1.docx]

**Supplemental Table 1** Characteristics of *Gallus gallus* gene-specific primers used for qPCR analysis in cecum mucosa

| Gene symbol | Forward (5` to 3`), Reverse (5` to 3`) | PCR product size (bp) | NCBI GenBank accession no. |
| --- | --- | --- | --- |
| *Reference genes* |  |  |  |
| *ACTB* | ATGAAGCCCAGAGCAAAAGA, GGGGTGTTGAAGGTCTCAAA | 223 | NM_205518.1 |
| *GAPDH* | ACTGTCAAGGCTGAGAACGG, AGCTGAGGGAGCTGAGATGA | 204 | NM_204305.1 |
| *SDHA* | ATTCCCGTTTTGCCTACGGT**,** GGGAGTTTGCTCCAAGACGA | 172 | NM_001277398.1 |
| *YWHAZ* | TTCCAACTTCCGTCTGCCTC**,** AGCAGTCTTCCTCGCTTGAC | 328 | NM_001031343.1 |
| *Target genes* |  |  |  |
| *CLDN3* | TTTCGGTCAGCGGGTTCCTC, CCGTCACGATGTTGTTGCCG | 171 | NM_204202.2 |
| *CLDN5* | ACTCATTGCAGGTCGCCAGA, AGCATCACGAGCGAGGGAAA | 147 | NM_204201.1 |
| *IL1b* | TCTGCCTGCAGAAGAAGCCT, CGCTGTCAGCAAAGTCCCTG | 168 | NM_204524 |
| *IL8L1* | GTGACACCCGGAAGAAACACT, CGTGCCTGAGCCATACCTTT | 109 | NM_205018.2 |
| *IL8L2* | AGGCACTTATGGCCAAGGCT, GGCACCGATGTGAAAGGTGG | 157 | NM_205498.2 |
| *JAM2* | GCTACATGAGGTGCGTGGGA, AAGGCTTGTGTCCTGTGCCA | 100 | XM_046907882.1 |
| *MUC2* | ATTCCTTGTGACGCTGGACC, GTGTGGGAGCAGTGGTTGAT | 190 | NM_001318434.1 |
| *MUC5ac* | ACATCACCCACAACACCGTT, TCTCCACCATCAAATCCAGGG | 158 | ENSGALG0000003414 |
| *MUC13* | GCATTCCTCAAGCAGAGGTG, CTCAGGCTGCCGTGATATTT | 73 | XM_015289971.1 |
| *OCLN* | CGACAGCATCACCGAGGACA, TTTGGTAGTCTGGGCTCCGC | 92 | NM_205128.1 |
| *TLR4* | GTCCGTGGCTGGAGGTCATC, AGGTCCAAGTCCAGGGTGGT | 103 | NM_001030693.2 |
| *VCAM1* | GGAGAAACCGCCACTGTCAT, GGCTCAAAGTCCATACCAGCA | 89 | XM_004936551.1 |

ACTB, β-actin; CLDN3, claudin 3; CLDN5, claudin 5; GAPDH, glyceraldehyde-3-phosphate dehydrogenase; IL1b, interleukin 1β; IL8L1, interleukin 8-like 1; IL8L2, interleukin 8-like2; JAM2; junctional adhesion molecule 2; MUC2, mucine 2; MUC5ac, mucine 5ac; MUC13, mucine 13; OCLN, occludin; SDAH, succinate dehydrogenase complex flavoprotein subunit A; TLR4, toll like receptor 4; VCAM1, vascular cell-adhesion molecule 1; YWHAZ, , tyrosine 3-monooxygenase/tryptophan 5-monooxygenase activation protein zeta.

**Supplemental Table 2** Characteristics of *Gallus gallus* gene-specific primers used for qPCR analysis for microarray validation

| Gene symbol | Forward (5` to 3`), Reverse (5` to 3`) | PCR product size (bp) | NCBI GenBank accession no. |
| --- | --- | --- | --- |
| *Reference genes* |  |  |  |
| *ACTB* | ATGAAGCCCAGAGCAAAAGA, GGGGTGTTGAAGGTCTCAAA | 223 | NM_205518.1 |
| *GAPDH* | ACTGTCAAGGCTGAGAACGG, AGCTGAGGGAGCTGAGATGA | 204 | NM_204305.1 |
| *SDHA* | ATTCCCGTTTTGCCTACGGT**,** GGGAGTTTGCTCCAAGACGA | 172 | NM_001277398.1 |
| *YWHAZ* | TTCCAACTTCCGTCTGCCTC**,** AGCAGTCTTCCTCGCTTGAC | 328 | NM_001031343.1 |
| *Target genes* |  |  |  |
| *ANGPTL2* | CCACAACCGCCAAGCAAAGT; GTAACGGCAGGCATGGTTGG | 134 | NM_001277699.2 |
| *C3AR1L* | CTGGACACAGGACCGATGGTT; AACCAACCCATTGCCTGGGA | 189 | NM_001030769.3 |
| *CCNB1* | ACTGGCTTGTCCAGGTCCAC; GCGCTGTTACACCCACCAAC | 135 | NM_001004369.2 |
| *FYB1* | GTGTGATTGGCACAGGGTGT; TGGTGTGCCCACATGTCTTG | 182 | NM_001031451.2 |
| *HTR7* | TCAGCTGCAGGGATGCATGA; GCCTGCTTCTTCCCATTGCTC | 151 | NM_001174134.2 |
| *KITLG* | TGAAGGGAGTTTGCACACGG; CTGTCACTGGATTCCCGCAG | 157 | NM_001105315.1 |
| *MYH1A* | CTGAGGCCCACTTCTCCCTG; TCCACCAGCAGAGGCAAACA | 161 | NM_001013396.2 |
| *PHC1* | GCAGTCTCCAGAAGTGCCAAG; AGAGTCTGAGGCAGGTGCTAC | 189 | NM_001006249.2 |
| *PLEK* | TGCGCATAAGGGAGGGCTAC; AGTGGTCCTGTTGCTTGGCT | 228 | NM_204863.2 |
| *PSTPIP2* | GCAACGCAAACCTGGTCACA; CTGATGCGCTCGCACTCTTG | 196 | XM_001233736.6 |
| *SMC2* | CCCTTAGTGGAGGTGCAAGC; TCTCCCACT GCT GC TTC AGA | 177 | NM_205230.2 |
| *TBXAS1* | TGCTCCTCCTGCGTCAACTC; CACCACAAACATCTGGCGACC | 227 | XM_040660825.2 |
| *TOP2A* | ATGATGCTGCCATCACCCTG; GGCCTGGCTTCAAACCATCA | 243 | NM_204791.3 |
| *TOPAZ1* | TTGCAGATGGAGTGGCTGCT; GGGCATGCAACGGCAATTCT | 117 | XM_046912383.1 |
| *TSHZ2* | CCAAACGGGCTGCAGGTTAC; CTGCTCAGGACCCACATCGT | 155 | XM_004947254.5 |
| *WWTR1* | CAGGTAGACTGCAGGCCCTC; CTGTCGCCTCCAGCTGATGA | 239 | XM_003641786.5 |
| *XCL1* | TCAGAAGGAGATCCTCGGGCT; GGTCATCCCTTGGTGCGAGT | 110 | NM_205046.2 |

ACTB, β-actin; ANGPTL2, angiopoietin-like 2; C3AR1L, complement C3a receptor 1 like; CCNB1, cyclin B1; FYB1, FYN binding protein 1; GAPDH, glyceraldehyde-3-phosphate dehydrogenase;; HTR7, 5-hydroxytryptamine (serotonin) receptor 7, adenylate cyclase coupled; KITLG, KIT ligand; MYH1A, myosin, heavy chain 1A skeletal muscle; PHC1, polyhomeotic homolog 1; PLEK, pleckstrin; PSTPIP2, proline-serine-threonine phosphatase interacting protein 2; SDAH, succinate dehydrogenase complex flavoprotein subunit A; SMC2, structural maintenance of chromosomes 2; TBXAS1, thromboxane A synthase 1; TOP2A, topoisomerase (DNA) II alpha; TOPAZ1; testis and ovary specific PAZ domain containing 1; TSHZ2, teashirt zinc finger homeobox 2; WWTR1, WW domain containing transcription regulator 1; XCL1, X-C motif chemokine ligand 1; YWHAZ, , tyrosine 3-monooxygenase/tryptophan 5-monooxygenase activation protein zeta.

**Supplemental Table 3** Characterization of *Pleurotus sapidus* mycelium

| Analyzed crude nutrient and energy content |  |
| --- | --- |
| Dry matter (% FM*) | 91.60 |
| CP (% DM) | 10.49 |
| EE (% DM) | 1.78 |
| CA (% DM) | 3.61 |
| CF (% DM) | 6.58 |
| Chitin (% DM) | 5.32 |
| Sugar (% DM) | 30.10 |
| Total glucans (% DM) | 36.60 |
| α-Glucans (% DM) | 5.43 |
| β-Glucans (% DM) | 31.17 |
| Gross energy (MJ/kg) | 17.69 |
| Calculated energy content |  |
| Metabolized energy (MJ/kg) | 7.75 |
| Amino acids, g/kg |  |
| Alanine | 11.86 |
| Arginine | 7.51 |
| Asparagine/aspartic acid | 8.97 |
| Cysteine | 1.67 |
| Glutamine/glutamic acid | 14.53 |
| Glycine | 4.78 |
| Histidine | 2.29 |
| Isoleucine | 4.15 |
| Leucine | 7.50 |
| Lysine | 6.44 |
| Methionine | 1.46 |
| Phenylalanine | 4.52 |
| Proline | 4.72 |
| Serine | 5.03 |
| Threonine | 4.99 |
| Tryptophan | 0.72 |
| Tyrosine | 2.90 |
| Valine | 4.79 |
| Fatty acids, % of total fatty acids˟ |  |
| C12:0 | 0.71 |
| C14:0 | 0.34 |
| C16:0 | 9.79 |
| C17:0 | 0.83 |
| C18:0 | 5.90 |
| C18:1 | 39.30 |
| C18:2 | 41.44 |
| C18:3 | 1.70 |

* FM, fresh matter after freeze-drying; Abbreviations: FM, fresh matter; DM, dry matter; CP, crude protein; EE, ether extract; CA, crude ash; CF, crude fiber

˟ only fatty acids >0.1 % of total fatty acids are shown

**Supplemental Table 4** relative abundance of bacterial taxa in cecum digesta of the broilers

|  | PSA-0 | PSA-2.5 | PSA-5.0 | *P* - value |
| --- | --- | --- | --- | --- |
| Phylum |  |  |  |  |
| Actinobacteriota | 0.0425 ± 0.0076 | 0.0464 ± 0.014 | 0.0354 ± 0.0122 | 0.120 |
| Firmicutes | 98.64 ± 0.5 | 98.6 ± 1 | 98.9 ± 0.9 | 0.120 |
| Proteobacteria | 1.31 ± 0.5 | 1.4 ± 0.97 | 1.01 ± 0.86 | 0.120 |
| Verrucomicrobiota | 0.00676 ± 0.00486 | 0.00586 ± 0.00501 | 0.00782 ± 0.00844 | 0.809 |
| Class |  |  |  |  |
| Bacilli | 11.64 ± 8.22 | 21.2 ± 15.6 | 15.3 ± 8.8 | 0.299 |
| Clostridia | 87 ± 8.25 | 77.3 ± 15.1 | 83.6 ± 8.4 | 0.299 |
| Coriobacteriia | 0.0425 ± 0.0076 | 0.0464 ± 0.014 | 0.0354 ± 0.0122 | 0.207 |
| Gammaproteobacteria | 1.31 ± 0.5 | 1.4 ± 0.97 | 1.01 ± 0.86 | 0.226 |
| Verrucomicrobiae | 0.00676 ± 0.00486 | 0.00586 ± 0.00501 | 0.00782 ± 0.00844 | 0.809 |
| Order |  |  |  |  |
| Christensenellales | 0.00361 ± 0.00441 | 0.00326 ± 0.00422 | 0.00378 ± 0.0051 | 0.998 |
| Clostridia UCG 014 | 0.383 ± 0.697^a^ | 0.00437 ± 0.01224^b^ | 0.258 ± 0.404^a^ | 0.035 |
| Clostridiales | 0.00485 ± 0.00871 | 0.0104 ± 0.0092 | 0.0099 ± 0.01042 | 0.260 |
| Coriobacteriales | 0.0425 ± 0.0076 | 0.0464 ± 0.014 | 0.0354 ± 0.0122 | 0.189 |
| Enterobacterales | 1.31 ± 0.5 | 1.4 ± 0.97 | 1.01 ± 0.86 | 0.253 |
| Erysipelotrichales | 2.01 ± 1.41 | 1.51 ± 0.65 | 2.12 ± 0.46 | 0.189 |
| Eubacteriales | 0.0146 ± 0.0074^c^ | 0.0208 ± 0.0078^b^ | 0.0318 ± 0.0095^a^ | 0.008 |
| Lachnospirales | 67.64 ± 9.3 | 60.2 ± 11.3 | 66.9 ± 7.2 | 0.298 |
| Lactobacillales | 9.63 ± 8.41 | 19.7 ± 15.8 | 13.2 ± 8.8 | 0.298 |
| Monoglobales | 0.657 ± 0.237 | 0.633 ± 0.34 | 0.5 ± 0.285 | 0.539 |
| Oscillospirales | 12.95 ± 3.12 | 11.4 ± 3.2 | 11.2 ± 2.4 | 0.539 |
| Peptococcales | 0.201 ± 0.162 | 0.293 ± 0.125 | 0.225 ± 0.167 | 0.260 |
| Peptostreptococcales Tissierellales | 5.14 ± 4.67 | 4.76 ± 5.86 | 4.41 ± 2.61 | 0.699 |
| Verrucomicrobiales | 0.00676 ± 0.00486 | 0.00586 ± 0.00501 | 0.00782 ± 0.00844 | 0.871 |
| Family |  |  |  |  |
| Akkermansiaceae | 0.00676 ± 0.00486 | 0.00586 ± 0.00501 | 0.00782 ± 0.00844 | 0.927 |
| Anaerofustaceae | 0.0146 ± 0.0074^c^ | 0.0208 ± 0.0078^b^ | 0.0318 ± 0.0095^a^ | 0.013 |
| Anaerovoracaceae | 0.0557 ± 0.0246 | 0.0654 ± 0.0276 | 0.0643 ± 0.0185 | 0.656 |
| Butyricicoccaceae | 3.02 ± 0.26 | 2.46 ± 0.73 | 2.39 ± 0.46 | 0.057 |
| Christensenellaceae | 0.00361 ± 0.00441 | 0.00326 ± 0.00422 | 0.00378 ± 0.0051 | 0.998 |
| Clostridia UCG 014 | 0.383 ± 0.697 | 0.00437 ± 0.01224 | 0.258 ± 0.404 | 0.057 |
| Clostridiaceae | 0.00485 ± 0.00871 | 0.0104 ± 0.0092 | 0.0099 ± 0.01042 | 0.340 |
| Clostridium methylpentosum group | 0.017 ± 0.0155 | 0.0185 ± 0.011 | 0.0156 ± 0.0167 | 0.713 |
| Eggerthellaceae | 0.0425 ± 0.0076 | 0.0464 ± 0.014 | 0.0354 ± 0.0122 | 0.191 |
| Enterobacteriaceae | 1.31 ± 0.5 | 1.4 ± 0.97 | 1.01 ± 0.86 | 0.340 |
| Enterococcaceae | 0.138 ± 0.164 | 0.0907 ± 0.0584 | 0.126 ± 0.102 | 0.656 |
| Erysipelatoclostridiaceae | 0.694 ± 0.396 | 0.599 ± 0.169 | 0.916 ± 0.301 | 0.058 |
| Erysipelotrichaceae | 1.31 ± 1.27 | 0.908 ± 0.567 | 1.21 ± 0.37 | 0.340 |
| Eubacterium coprostanoligenes group | 0.969 ± 0.379 | 1 ± 0.43 | 1.14 ± 0.37 | 0.656 |
| Lachnospiraceae | 67.64 ± 9.3 | 60.2 ± 11.3 | 66.9 ± 7.2 | 0.340 |
| Lactobacillaceae | 9.48 ± 8.48 | 19.6 ± 15.8 | 13.1 ± 8.9 | 0.340 |
| Monoglobaceae | 0.657 ± 0.237 | 0.633 ± 0.34 | 0.5 ± 0.285 | 0.656 |
| Oscillospiraceae | 3.4 ± 1.59 | 3.25 ± 1.44 | 2.96 ± 0.91 | 0.927 |
| Oscillospirales | 0.156 ± 0.139 | 0.199 ± 0.124 | 0.157 ± 0.169 | 0.656 |
| Peptococcaceae | 0.201 ± 0.162 | 0.293 ± 0.125 | 0.225 ± 0.167 | 0.340 |
| Peptostreptococcaceae | 5.09 ± 4.67 | 4.7 ± 5.86 | 4.34 ± 2.62 | 0.713 |
| Ruminococcaceae | 5.4 ± 1.49 | 4.48 ± 1.05 | 4.58 ± 1.22 | 0.340 |
| Streptococcaceae | 0.0106 ± 0.0192 | 0.00883 ± 0.01227 | 0.0077 ± 0.00891 | 0.927 |
| Genus |  |  |  |  |
| *Akkermansia* | 0.00676 ± 0.00486 | 0.00586 ± 0.00501 | 0.00782 ± 0.00844 | 0.906 |
| *Anaerofustis* | 0.0146 ± 0.0074^c^ | 0.0208 ± 0.0078^b^ | 0.0318 ± 0.0095^a^ | 0.032 |
| *Anaerostipes* | 2.82 ± 1.61 | 2.94 ± 2.9 | 4.82 ± 2.52 | 0.178 |
| *Anaerotruncus* | 0.284 ± 0.16 | 0.279 ± 0.14 | 0.252 ± 0.123 | 0.963 |
| *ASF356* | 0.212 ± 0.081 | 0.22 ± 0.082 | 0.205 ± 0.072 | 0.963 |
| *Blautia* | 6.96 ± 3.16 | 5.2 ± 4.47 | 7.51 ± 2.99 | 0.297 |
| *Butyricicoccus* | 2.88 ± 0.25 | 2.36 ± 0.73 | 2.21 ± 0.41 | 0.092 |
| *Caproiciproducens* | 0.0923 ± 0.0541 | 0.0874 ± 0.0472 | 0.108 ± 0.036 | 0.826 |
| *CHKCI001* | 0.357 ± 0.077 | 0.329 ± 0.061 | 0.403 ± 0.091 | 0.217 |
| *Christensenellaceae R 7 group* | 0.00361 ± 0.00441 | 0.00326 ± 0.00422 | 0.00378 ± 0.0051 | 0.998 |
| *Clostridia UCG 014* | 0.383 ± 0.697 | 0.00437 ± 0.01224 | 0.258 ± 0.404 | 0.092 |
| *Clostridioides* | 0.113 ± 0.249 | 0.198 ± 0.323 | 0.0112 ± 0.0171 | 0.131 |
| *Clostridium innocuum group* | 0.141 ± 0.04 | 0.15 ± 0.057 | 0.123 ± 0.043 | 0.449 |
| *Clostridium methylpentosum group* | 0.017 ± 0.0155 | 0.0185 ± 0.011 | 0.0156 ± 0.0167 | 0.826 |
| *Clostridium sensu stricto 1* | 0.00485 ± 0.00871 | 0.0104 ± 0.0092 | 0.0099 ± 0.01042 | 0.297 |
| *Colidextribacter* | 2.09 ± 1.16 | 2.21 ± 1.21 | 2.02 ± 0.73 | 0.931 |
| *DTU089* | 0.544 ± 0.296 | 0.468 ± 0.162 | 0.428 ± 0.131 | 0.906 |
| *Eggerthella* | 0.0237 ± 0.0098 | 0.0215 ± 0.0099 | 0.0174 ± 0.0071 | 0.378 |
| *Eisenbergiella* | 3.92 ± 0.83 | 4.01 ± 1.27 | 4.22 ± 1.34 | 0.906 |
| *Enterococcus* | 0.138 ± 0.164 | 0.0907 ± 0.0584 | 0.126 ± 0.102 | 0.733 |
| *Erysipelatoclostridium* | 0.694 ± 0.396 | 0.599 ± 0.169 | 0.916 ± 0.301 | 0.095 |
| *Escherichia Shigella* | 1.28 ± 0.48 | 1.39 ± 0.96 | 1.01 ± 0.86 | 0.297 |
| *Eubacterium coprostanoligenes group* | 0.969 ± 0.379 | 1 ± 0.43 | 1.14 ± 0.37 | 0.692 |
| *Eubacterium hallii group* | 3.1 ± 1.01 | 3.32 ± 0.83 | 2.7 ± 1.02 | 0.378 |
| *Eubacterium nodatum group* | 0.0557 ± 0.0246 | 0.0654 ± 0.0276 | 0.0643 ± 0.0185 | 0.729 |
| *Faecalibacterium* | 0.00357 ± 0.00517 | 0.00242 ± 0.00395 | 0.00526 ± 0.00681 | 0.826 |
| *Flavonifractor* | 0.509 ± 0.235 | 0.393 ± 0.144 | 0.373 ± 0.115 | 0.297 |
| *GCA 900066575* | 1.38 ± 0.7 | 2.32 ± 1.13 | 1.6 ± 1.64 | 0.192 |
| *Gordonibacter* | 0.0188 ± 0.005 | 0.025 ± 0.0079 | 0.018 ± 0.0087 | 0.189 |
| *Hydrogenoanaerobacterium* | 0.156 ± 0.139 | 0.199 ± 0.124 | 0.157 ± 0.169 | 0.729 |
| *Incertae Sedis* | 3.27 ± 1.1 | 2.3 ± 0.55 | 2.44 ± 0.74 | 0.192 |
| *Klebsiella* | 0.0277 ± 0.0336 | 0.00736 ± 0.01062 | 0.000761 ± 0.002635 | 0.095 |
| *Lachnoclostridium* | 1.38 ± 0.45 | 1.5 ± 0.46 | 1.33 ± 0.49 | 0.826 |
| *Lachnospiraceae UCG 010* | 0.0526 ± 0.0325 | 0.0535 ± 0.0384 | 0.0488 ± 0.0538 | 0.902 |
| *Lactobacillus* | 9.48 ± 8.48 | 19.6 ± 15.8 | 13.1 ± 8.9 | 0.378 |
| *Monoglobus* | 0.657 ± 0.237 | 0.633 ± 0.34 | 0.5 ± 0.285 | 0.697 |
| *Oscillibacter* | 0.321 ± 0.201 | 0.24 ± 0.152 | 0.207 ± 0.182 | 0.480 |
| *Paludicola* | 0.166 ± 0.117 | 0.207 ± 0.116 | 0.214 ± 0.173 | 0.826 |
| *Romboutsia* | 4.43 ± 4.64 | 4.42 ± 5.9 | 3.86 ± 2.78 | 0.845 |
| *Ruminococcus torques group* | 13.67 ± 4.77 | 9.04 ± 3.63 | 11.4 ± 3.9 | 0.178 |
| *Sellimonas* | 4.13 ± 0.94 | 3.03 ± 0.73 | 3.77 ± 0.68 | 0.095 |
| *Shuttleworthia* | 1.09 ± 0.47 | 1.05 ± 0.46 | 0.849 ± 0.45 | 0.697 |
| *Streptococcus* | 0.0106 ± 0.0192 | 0.00883 ± 0.01227 | 0.0077 ± 0.00891 | 0.945 |
| *Turicibacter* | 0.48 ± 1.164 | 0.183 ± 0.391 | 0.182 ± 0.173 | 0.378 |
| *Tyzzerella* | 0.0996 ± 0.0641 | 0.119 ± 0.059 | 0.103 ± 0.038 | 0.906 |
| *UBA1819* | 0.0087 ± 0.01993 | 0.0135 ± 0.0341 | 0.0462 ± 0.0499 | 0.178 |
| *UCG 009* | 0.136 ± 0.066 | 0.107 ± 0.071 | 0.175 ± 0.112 | 0.297 |
| *uncultured Erysipelotrichaceae* | 0.693 ± 0.203 | 0.575 ± 0.315 | 0.903 ± 0.274 | 0.131 |
| *uncultured Lachnospiraceae* | 0.603 ± 0.515 | 0.394 ± 0.454 | 0.747 ± 0.6 | 0.394 |
| *uncultured Oscillospiraceae* | 0.325 ± 0.155 | 0.287 ± 0.077 | 0.267 ± 0.051 | 0.826 |
| *uncultured Peptococcaceae* | 0.201 ± 0.162 | 0.293 ± 0.125 | 0.225 ± 0.167 | 0.316 |
| *uncultured Ruminococcaceae* | 0.532 ± 0.08 | 0.436 ± 0.121 | 0.426 ± 0.13 | 0.192 |
| *unknown Lachnospiraceae* | 27.86 ± 5.72 | 26.7 ± 7.4 | 27.3 ± 4.9 | 0.954 |
| *unknown Oscillospiraceae* | 0.157 ± 0.136 | 0.121 ± 0.093 | 0.0935 ± 0.0697 | 0.845 |
| *unknown Peptostreptococcaceae* | 0.548 ± 0.822 | 0.0843 ± 0.1044 | 0.47 ± 0.476 | 0.131 |
| *unknown Ruminococcaceae* | 0.502 ± 0.217 | 0.685 ± 0.397 | 0.666 ± 0.386 | 0.692 |
| Species |  |  |  |  |
| *Anaerofustis stercorihominis* | 0.0146 ± 0.0074^c^ | 0.0208 ± 0.0078^b^ | 0.0318 ± 0.0095^a^ | < 0.001 |
| *Anaerostipes butyraticus* | 2.71 ± 1.64 | 2.78 ± 2.94 | 4.61 ± 2.65 | 0.184 |
| *bacterium ic1311* | 0.603 ± 0.515 | 0.394 ± 0.454 | 0.747 ± 0.6 | 0.426 |
| *Blautia hydrogenotrophica* | 0.265 ± 0.155 | 0.288 ± 0.153 | 0.328 ± 0.277 | 0.997 |
| *Clostridiales bacterium 1* | 0.693 ± 0.203 | 0.575 ± 0.315 | 0.903 ± 0.274 | 0.121 |
| *Clostridioides difficile* | 0.113 ± 0.249 | 0.198 ± 0.323 | 0.0112 ± 0.0171 | 0.121 |
| *Clostridium aldenense* | 0.0314 ± 0.0241 | 0.0372 ± 0.022 | 0.0465 ± 0.0318 | 0.735 |
| *Clostridium leptum* | 0.00765 ± 0.00698^c^ | 0.031 ± 0.0076^b^ | 0.046 ± 0.0111^a^ | 0.018 |
| *Clostridium methylpentosum* | 0.017 ± 0.0155 | 0.0185 ± 0.011 | 0.0156 ± 0.0167 | 0.854 |
| *Clostridium paraputrificum* | 0.00485 ± 0.00871 | 0.0104 ± 0.0092 | 0.0099 ± 0.01042 | 0.346 |
| *Clostridium spiroforme* | 0.0982 ± 0.076 | 0.0833 ± 0.0635 | 0.13 ± 0.085 | 0.599 |
| *Eubacteriaceae bacterium* | 0.0129 ± 0.0091 | 0.022 ± 0.0105 | 0.0255 ± 0.0103 | 0.094 |
| *Gordonibacter pamelaeae* | 0.0188 ± 0.005 | 0.025 ± 0.0079 | 0.018 ± 0.0087 | 0.190 |
| *Ihubacter massiliensis* | 0.0557 ± 0.0246 | 0.0654 ± 0.0276 | 0.0643 ± 0.0185 | 0.735 |
| *Lachnoclostridium phocaeense* | 1.35 ± 0.22 | 1.01 ± 0.33 | 1.29 ± 0.44 | 0.190 |
| *Lactobacillus johnsonii* | 2.07 ± 1.58 | 1.85 ± 2.22 | 1.72 ± 1.64 | 0.912 |
| *Lactobacillus reuteri* | 0.56 ± 0.749 | 0.758 ± 0.856 | 0.627 ± 0.599 | 0.906 |
| *Massiliomicrobiota timonensis* | 0.581 ± 0.351 | 0.496 ± 0.207 | 0.76 ± 0.244 | 0.184 |
| *metagenome Ruminococcaceae* | 0.282 ± 0.045^a^ | 0.177 ± 0.07^b^ | 0.186 ± 0.071^b^ | 0.037 |
| *Ruminococcus sp* | 0.00966 ± 0.02007 | 0.0159 ± 0.0298 | 0.0263 ± 0.0211 | 0.228 |
| *Streptococcus pluranimalium* | 0.0106 ± 0.0192 | 0.00883 ± 0.01227 | 0.0077 ± 0.00891 | 0.963 |
| *uncultured bacterium Akkermansia* | 0.00676 ± 0.00486 | 0.00586 ± 0.00501 | 0.00782 ± 0.00844 | 0.930 |
| *uncultured bacterium ASF356* | 0.212 ± 0.081 | 0.22 ± 0.082 | 0.205 ± 0.072 | 0.970 |
| *uncultured bacterium Butyricicoccus* | 0.23 ± 0.377 | 0.548 ± 0.51 | 0.446 ± 0.511 | 0.376 |
| *uncultured bacterium Caproiciproducens* | 0.0923 ± 0.0541 | 0.0874 ± 0.0472 | 0.108 ± 0.036 | 0.854 |
| *uncultured bacterium Eggerthella* | 0.0237 ± 0.0098 | 0.0215 ± 0.0099 | 0.0174 ± 0.0071 | 0.417 |
| *uncultured bacterium Erysipelatoclostridium* | 0 ± 0 | 0.00558 ± 0.00875 | 0.0159 ± 0.0161 | 0.081 |
| *uncultured bacterium Eubacterium hallii group* | 3.1 ± 1.01 | 3.32 ± 0.83 | 2.7 ± 1.02 | 0.401 |
| *uncultured bacterium Flavonifractor* | 0.47 ± 0.244 | 0.354 ± 0.151 | 0.346 ± 0.109 | 0.401 |
| *uncultured bacterium Hydrogenoanaerobacterium* | 0.156 ± 0.139 | 0.199 ± 0.124 | 0.157 ± 0.169 | 0.735 |
| *uncultured bacterium Incertae Sedis* | 1.35 ± 0.56^a^ | 0.552 ± 0.27^b^ | 0.607 ± 0.312^b^ | 0.018 |
| *uncultured bacterium Lachnospiraceae UCG 010* | 0.0526 ± 0.0325 | 0.0535 ± 0.0384 | 0.0488 ± 0.0538 | 0.926 |
| *uncultured bacterium Monoglobus* | 0.657 ± 0.237 | 0.633 ± 0.34 | 0.5 ± 0.285 | 0.735 |
| *uncultured bacterium Paludicola* | 0.0893 ± 0.0653 | 0.0826 ± 0.049 | 0.0598 ± 0.0499 | 0.735 |
| *uncultured bacterium Peptococcaceae* | 0.201 ± 0.162 | 0.293 ± 0.125 | 0.225 ± 0.167 | 0.371 |
| *uncultured bacterium Ruminococcus torques group* | 5.35 ± 1.2 | 4.98 ± 1.14 | 5.52 ± 1.02 | 0.735 |
| *uncultured bacterium Sellimonas* | 0.741 ± 0.998 | 0.595 ± 0.553 | 0.74 ± 0.71 | 0.963 |
| *uncultured bacterium Shuttleworthia* | 1.09 ± 0.47 | 1.05 ± 0.46 | 0.849 ± 0.45 | 0.735 |
| *uncultured bacterium Tyzzerella* | 0.0996 ± 0.0641 | 0.119 ± 0.059 | 0.103 ± 0.038 | 0.930 |
| *uncultured Clostridiales* | 8.31 ± 4.93 | 4.04 ± 3.47 | 5.82 ± 3.56 | 0.121 |
| *uncultured organism Anaerotruncus* | 0.284 ± 0.16 | 0.279 ± 0.14 | 0.252 ± 0.123 | 0.970 |
| *uncultured organism Butyricicoccus* | 0.834 ± 0.194 | 0.587 ± 0.192 | 0.576 ± 0.254 | 0.087 |
| *uncultured organism CHKCI001* | 0.357 ± 0.077 | 0.329 ± 0.061 | 0.403 ± 0.091 | 0.229 |
| *uncultured organism Incertae Sedis* | 1.08 ± 0.64 | 0.887 ± 0.268 | 0.964 ± 0.327 | 0.930 |
| *uncultured organism Lachnoclostridium* | 0.586 ± 0.218 | 0.505 ± 0.199 | 0.559 ± 0.246 | 0.914 |
| *uncultured organism Oscillibacter* | 0.308 ± 0.193 | 0.22 ± 0.141 | 0.194 ± 0.18 | 0.422 |
| *uncultured organism Sellimonas* | 0.077 ± 0.0758 | 0.176 ± 0.132 | 0.104 ± 0.087 | 0.298 |
| *uncultured organism UBA1819* | 0.0087 ± 0.01993 | 0.0135 ± 0.0341 | 0.0462 ± 0.0499 | 0.184 |
| *uncultured Oscillospiraceae* | 0.325 ± 0.155 | 0.287 ± 0.077 | 0.267 ± 0.051 | 0.854 |
| *unknown Anaerostipes* | 0.119 ± 0.054 | 0.157 ± 0.105 | 0.208 ± 0.184 | 0.963 |
| *unknown Blautia* | 6.7 ± 3.23 | 4.91 ± 4.46 | 7.18 ± 3.15 | 0.376 |
| *unknown Butyricicoccus* | 1.81 ± 0.34^a^ | 1.22 ± 0.38^b^ | 1.19 ± 0.45^b^ | 0.018 |
| *unknown Christensenellaceae R 7 group* | 0.00361 ± 0.00441 | 0.00326 ± 0.00422 | 0.00378 ± 0.0051 | 0.998 |
| *unknown Clostridia UCG 014* | 0.383 ± 0.697 | 0.00437 ± 0.01224 | 0.258 ± 0.404 | 0.066 |
| *unknown Clostridium innocuum group* | 0.141 ± 0.04 | 0.15 ± 0.057 | 0.123 ± 0.043 | 0.490 |
| *unknown Colidextribacter* | 2.09 ± 1.16 | 2.21 ± 1.21 | 2.02 ± 0.73 | 0.956 |
| *unknown DTU089* | 0.544 ± 0.296 | 0.468 ± 0.162 | 0.428 ± 0.131 | 0.930 |
| *unknown Eisenbergiella* | 3.92 ± 0.83 | 4.01 ± 1.27 | 4.22 ± 1.34 | 0.930 |
| *unknown Enterococcus* | 0.138 ± 0.164 | 0.0907 ± 0.0584 | 0.126 ± 0.102 | 0.746 |
| *unknown Erysipelatoclostridium* | 0.0144 ± 0.0091 | 0.0138 ± 0.0092 | 0.01 ± 0.0105 | 0.735 |
| *unknown Escherichia Shigella* | 1.28 ± 0.48 | 1.39 ± 0.96 | 1.01 ± 0.86 | 0.314 |
| *unknown Eubacterium coprostanoligenes group* | 0.956 ± 0.377 | 0.983 ± 0.433 | 1.11 ± 0.37 | 0.735 |
| *unknown Faecalibacterium* | 0.00357 ± 0.00517 | 0.00242 ± 0.00395 | 0.00526 ± 0.00681 | 0.857 |
| *unknown Flavonifractor* | 0.0394 ± 0.0371 | 0.039 ± 0.0368 | 0.0263 ± 0.0215 | 0.914 |
| *unknown GCA 900066575* | 1.38 ± 0.7 | 2.32 ± 1.13 | 1.6 ± 1.64 | 0.206 |
| *unknown Incertae Sedis* | 0.828 ± 0.526 | 0.831 ± 0.369 | 0.819 ± 0.334 | 0.970 |
| *unknown Klebsiella* | 0.0277 ± 0.0336 | 0.00736 ± 0.01062 | 0.000761 ± 0.002635 | 0.087 |
| *unknown Lachnoclostridium* | 0.767 ± 0.412 | 0.954 ± 0.394 | 0.722 ± 0.317 | 0.599 |
| *unknown Lachnospiraceae* | 27.86 ± 5.72 | 26.7 ± 7.4 | 27.3 ± 4.9 | 0.963 |
| *unknown Lactobacillus* | 6.85 ± 7.94 | 17 ± 16.4 | 10.7 ± 9.4 | 0.401 |
| *unknown Oscillibacter* | 0.0129 ± 0.0143 | 0.0194 ± 0.0148 | 0.0131 ± 0.0081 | 0.771 |
| *unknown Oscillospiraceae* | 0.157 ± 0.136 | 0.121 ± 0.093 | 0.0935 ± 0.0697 | 0.906 |
| *unknown Paludicola* | 0.0764 ± 0.058 | 0.125 ± 0.089 | 0.154 ± 0.134 | 0.587 |
| *unknown Peptostreptococcaceae* | 0.548 ± 0.822 | 0.0843 ± 0.1044 | 0.47 ± 0.476 | 0.121 |
| *unknown Romboutsia* | 4.43 ± 4.64 | 4.42 ± 5.9 | 3.86 ± 2.78 | 0.906 |
| *unknown Ruminococcaceae* | 0.502 ± 0.217 | 0.685 ± 0.397 | 0.666 ± 0.386 | 0.735 |
| *unknown Sellimonas* | 1.96 ± 0.86 | 1.24 ± 0.63 | 1.64 ± 0.71 | 0.191 |
| *unknown Turicibacter* | 0.48 ± 1.164 | 0.183 ± 0.391 | 0.182 ± 0.173 | 0.401 |
| *unknown UCG 009* | 0.136 ± 0.066 | 0.107 ± 0.071 | 0.175 ± 0.112 | 0.346 |
| *unknown uncultured Ruminococcaceae* | 0.251 ± 0.091 | 0.258 ± 0.083 | 0.240 ± 0.096 | 0.930 |

Data are means ± SD for *n* = 12 broilers/group. ^a,b^ means without a common letter differ across the groups.

**Supplemental Table 5** up- and downregulated genes in liver after filtering (*P* < 0.05 and FC < -1.3; FC > 1.3) comparing group PSA-5.0 vs. group PSA-0

| Gene symbol | Sig log ratio | Fold change | *P* - value |
| --- | --- | --- | --- |
| TOPAZ1 | 0.98 | 1.97 | 0.023 |
| MYH1A | 0.70 | 1.63 | 0.041 |
| LOC429206 | 0.67 | 1.59 | 0.025 |
| XCL1 | 0.66 | 1.58 | 0.013 |
| LOC101750249 | 0.64 | 1.56 | 0.014 |
| C3AR1 | 0.63 | 1.55 | 0.007 |
| LOC422924 | 0.63 | 1.55 | 0.018 |
| GJD2 | 0.63 | 1.55 | 0.007 |
| ASL1 | 0.61 | 1.53 | 0.048 |
| PLACL2 | 0.61 | 1.53 | 0.022 |
| MIR205B | 0.60 | 1.52 | 0.034 |
| SPI1 | 0.59 | 1.50 | 0.021 |
| CRNN | 0.57 | 1.48 | 0.029 |
| PSTPIP2 | 0.55 | 1.47 | 0.006 |
| MIR1602 | 0.54 | 1.46 | 0.003 |
| RHCE | 0.54 | 1.45 | 0.030 |
| SLC1A6 | 0.53 | 1.44 | 0.036 |
| USP18 | 0.52 | 1.44 | 0.043 |
| GLRA3 | 0.52 | 1.43 | 0.012 |
| VWC2 | 0.52 | 1.43 | 0.012 |
| K123 | 0.51 | 1.43 | 0.035 |
| C28H19orf35 | 0.51 | 1.42 | 0.046 |
| IRX6 | 0.50 | 1.42 | 0.021 |
| TBXAS1 | 0.50 | 1.41 | 0.011 |
| LOC101749756 | 0.49 | 1.40 | 0.046 |
| OR12D2 | 0.49 | 1.40 | 0.009 |
| GPR35 | 0.48 | 1.40 | 0.042 |
| FUT10 | 0.48 | 1.39 | 0.005 |
| MYL10 | 0.48 | 1.39 | 0.031 |
| BPI | 0.48 | 1.39 | 0.031 |
| SAMD9L | 0.48 | 1.39 | 0.043 |
| MMRN1 | 0.47 | 1.39 | 0.016 |
| LOC100857124 | 0.47 | 1.39 | 0.042 |
| LOC420734 | 0.47 | 1.39 | 0.020 |
| GRIA4 | 0.47 | 1.38 | 0.043 |
| LOC101750621 | 0.47 | 1.38 | 0.014 |
| C1QC | 0.46 | 1.38 | 0.023 |
| ADCYAP1 | 0.46 | 1.38 | 0.039 |
| TLR2-1 | 0.46 | 1.38 | 0.000 |
| IGSF10 | 0.46 | 1.38 | 0.009 |
| LOC100859916 | 0.46 | 1.37 | 0.015 |
| TNIP3 | 0.46 | 1.37 | 0.033 |
| NPY5R | 0.45 | 1.37 | 0.026 |
| LOC771494 | 0.45 | 1.37 | 0.025 |
| LOC419677 | 0.45 | 1.37 | 0.034 |
| HTR7 | 0.45 | 1.37 | 0.010 |
| VSIG4 | 0.45 | 1.37 | 0.006 |
| RIMS1 | 0.45 | 1.37 | 0.007 |
| XCR1 | 0.45 | 1.37 | 0.050 |
| KCNJ4 | 0.45 | 1.36 | 0.028 |
| LOC100859916 | 0.45 | 1.36 | 0.048 |
| ZNF831 | 0.45 | 1.36 | 0.043 |
| NIM1K | 0.44 | 1.36 | 0.026 |
| RASGRF2 | 0.44 | 1.35 | 0.044 |
| SPOCK3 | 0.44 | 1.35 | 0.028 |
| RBM20 | 0.44 | 1.35 | 0.039 |
| SPTLC3 | 0.44 | 1.35 | 0.013 |
| GPR132 | 0.43 | 1.35 | 0.048 |
| LOC422895 | 0.43 | 1.35 | 0.044 |
| SUSD3 | 0.43 | 1.35 | 0.012 |
| CSF2RB | 0.42 | 1.34 | 0.012 |
| LEKR1 | 0.42 | 1.34 | 0.017 |
| C1ORF94 | 0.42 | 1.34 | 0.027 |
| LOC423629 | 0.42 | 1.34 | 0.005 |
| TRPM3 | 0.42 | 1.33 | 0.034 |
| GNA14 | 0.41 | 1.33 | 0.022 |
| DDX4 | 0.41 | 1.33 | 0.041 |
| LOC101749988 | 0.41 | 1.33 | 0.044 |
| MEPE | 0.41 | 1.33 | 0.020 |
| CYBB | 0.41 | 1.33 | 0.020 |
| TMEM132B | 0.41 | 1.33 | 0.043 |
| ATP8A2 | 0.41 | 1.33 | 0.045 |
| SLC28A3 | 0.41 | 1.32 | 0.012 |
| LOC416309 | 0.41 | 1.32 | 0.016 |
| HTR6 | 0.40 | 1.32 | 0.040 |
| CDH19 | 0.40 | 1.32 | 0.014 |
| CYSLTR2 | 0.40 | 1.32 | 0.025 |
| PTPRC | 0.40 | 1.32 | 0.004 |
| LOC101749876 | 0.40 | 1.32 | 0.015 |
| PLEK | 0.40 | 1.32 | 0.005 |
| GALR1 | 0.40 | 1.32 | 0.020 |
| CRISP2 | 0.40 | 1.32 | 0.037 |
| LOC101750051 | 0.40 | 1.32 | 0.031 |
| LOC770026 | 0.40 | 1.32 | 0.049 |
| MC2R | 0.40 | 1.32 | 0.029 |
| VTCN1 | 0.40 | 1.32 | 0.005 |
| TECTB | 0.39 | 1.31 | 0.020 |
| LYPD6 | 0.39 | 1.31 | 0.030 |
| PIK3CD | 0.39 | 1.31 | 0.009 |
| KCNA2 | 0.39 | 1.31 | 0.019 |
| SMPX | 0.39 | 1.31 | 0.022 |
| FYB | 0.39 | 1.31 | 0.003 |
| CPA6 | 0.39 | 1.31 | 0.047 |
| ACTL9 | 0.38 | 1.31 | 0.038 |
| CLCC1 | -0.39 | -1.31 | 0.037 |
| SRGAP1 | -0.39 | -1.31 | 0.046 |
| LOC421106 | -0.40 | -1.32 | 0.006 |
| KITLG | -0.40 | -1.32 | 0.020 |
| MKI67 | -0.40 | -1.32 | 0.013 |
| RRM2 | -0.40 | -1.32 | 0.032 |
| KCNIP4 | -0.40 | -1.32 | 0.049 |
| MAP1B | -0.40 | -1.32 | 0.040 |
| PTPRF | -0.41 | -1.33 | 0.048 |
| CD99 | -0.41 | -1.33 | 0.043 |
| RIPK4 | -0.42 | -1.33 | 0.042 |
| LMO4 | -0.42 | -1.34 | 0.022 |
| TSHZ2 | -0.42 | -1.34 | 0.035 |
| TTK | -0.42 | -1.34 | 0.046 |
| CENPK | -0.42 | -1.34 | 0.013 |
| CASC5 | -0.43 | -1.35 | 0.010 |
| MAL2 | -0.44 | -1.36 | 0.009 |
| CCNA2 | -0.44 | -1.36 | 0.047 |
| 45179 | -0.45 | -1.36 | 0.044 |
| COL6A2 | -0.45 | -1.37 | 0.040 |
| SLITRK2 | -0.45 | -1.37 | 0.041 |
| COL6A1 | -0.45 | -1.37 | 0.031 |
| SMAD9 | -0.46 | -1.37 | 0.045 |
| GNAI1 | -0.47 | -1.38 | 0.034 |
| TTC6 | -0.47 | -1.39 | 0.017 |
| PHC1 | -0.47 | -1.39 | 0.020 |
| WWTR1 | -0.48 | -1.39 | 0.025 |
| TACC3 | -0.48 | -1.40 | 0.049 |
| SMC2 | -0.48 | -1.40 | 0.026 |
| SHANK2 | -0.48 | -1.40 | 0.036 |
| LOC423793 | -0.49 | -1.40 | 0.004 |
| MKI67 | -0.49 | -1.41 | 0.019 |
| CCNB2 | -0.50 | -1.42 | 0.015 |
| SPRY4 | -0.51 | -1.42 | 0.032 |
| SFXN5 | -0.51 | -1.42 | 0.037 |
| PTN | -0.51 | -1.43 | 0.012 |
| HHIP | -0.52 | -1.43 | 0.012 |
| LOC423499 | -0.52 | -1.43 | 0.017 |
| CTNNB1 | -0.52 | -1.44 | 0.017 |
| TOP2A | -0.54 | -1.45 | 0.008 |
| PXDN | -0.54 | -1.46 | 0.012 |
| SEMA5A | -0.58 | -1.49 | 0.041 |
| FGL1 | -0.59 | -1.51 | 0.036 |
| RNF138 | -0.60 | -1.51 | 0.040 |
| CAMK1D | -0.62 | -1.54 | 0.046 |
| NSMF | -0.66 | -1.58 | 0.023 |
| ANGPTL2 | -0.66 | -1.58 | 0.032 |
| MXRA8 | -0.72 | -1.64 | 0.008 |
| B3GALT5 | -0.75 | -1.68 | 0.018 |
| KIF20B | -0.79 | -1.73 | 0.003 |

FCs were calculated from the signal log ratios, which were calculated from *n* = 6 microarrays/group. Abbreviations: FC, fold change

**Supplemental Table 6** up- and downregulated genes in liver after filtering (*P* < 0.05 and FC < -1.3; FC > 1.3) comparing group PSA-2.5 vs. group PSA-0.

| Gene symbol | Sig log ratio | Fold change | *P* - value |
| --- | --- | --- | --- |
| GUCY2C | 1.18 | 2.27 | 0.004 |
| LOC422513 | 0.98 | 1.97 | 0.017 |
| TMEM27 | 0.83 | 1.78 | 0.003 |
| ACE2 | 0.76 | 1.69 | 0.004 |
| C11ORF34 | 0.73 | 1.66 | 0.017 |
| LOC100859846 | 0.72 | 1.65 | 0.003 |
| DNAJC6 | 0.71 | 1.64 | 0.024 |
| CYP2K1L | 0.69 | 1.61 | 0.028 |
| OVSTL | 0.65 | 1.57 | 0.007 |
| LOC428289 | 0.58 | 1.49 | 0.037 |
| SSTR1 | 0.56 | 1.47 | 0.022 |
| AQP11 | 0.55 | 1.46 | 0.041 |
| KCNJ16 | 0.54 | 1.45 | 0.050 |
| FRMPD2 | 0.53 | 1.45 | 0.044 |
| FMO3 | 0.53 | 1.44 | 0.015 |
| STARD4 | 0.52 | 1.43 | 0.027 |
| DHCR7 | 0.51 | 1.43 | 0.031 |
| IL22RA1 | 0.51 | 1.42 | 0.025 |
| GSTA | 0.51 | 1.42 | 0.025 |
| ARRDC2 | 0.49 | 1.40 | 0.034 |
| LNX2 | 0.48 | 1.39 | 0.002 |
| AGXT2 | 0.47 | 1.38 | 0.010 |
| PIGP | 0.46 | 1.38 | 0.032 |
| MKI67 | 0.45 | 1.37 | 0.033 |
| EMB | 0.43 | 1.35 | 0.015 |
| ACAD10 | 0.43 | 1.35 | 0.024 |
| RHOU | 0.43 | 1.34 | 0.040 |
| SRD5A2 | 0.40 | 1.32 | 0.022 |
| SEC24A | 0.40 | 1.32 | 0.002 |
| PTTG1 | 0.40 | 1.32 | 0.044 |
| NMI | 0.39 | 1.31 | 0.032 |
| MANBA | 0.39 | 1.31 | 0.010 |
| DHRS3 | 0.39 | 1.31 | 0.010 |
| LOC420552 | -0.39 | -1.31 | 0.006 |
| ARID3B | -0.41 | -1.32 | 0.009 |
| SLC19A2 | -0.41 | -1.33 | 0.010 |
| AXIN2 | -0.42 | -1.34 | 0.029 |
| TFRC | -0.42 | -1.34 | 0.006 |
| NTNG1 | -0.43 | -1.35 | 0.043 |
| LOC417386 | -0.44 | -1.35 | 0.014 |
| MIR181A1 | -0.44 | -1.35 | 0.015 |
| GALK1 | -0.44 | -1.36 | 0.033 |
| PPA1 | -0.45 | -1.37 | 0.020 |
| HADHA | -0.45 | -1.37 | 0.016 |
| RBP | -0.46 | -1.37 | 0.007 |
| LOC101751093 | -0.46 | -1.37 | 0.038 |
| MIR16C | -0.46 | -1.38 | 0.011 |
| RP11-292K15.2 | -0.51 | -1.42 | 0.028 |
| TMEM186 | -0.51 | -1.43 | 0.011 |
| GAL2 | -0.52 | -1.44 | 0.002 |
| SLC16A1 | -0.52 | -1.44 | 0.040 |
| ACAA1 | -0.53 | -1.44 | 0.042 |
| MIR6578 | -0.63 | -1.54 | 0.017 |
| LOC768786 | -0.67 | -1.59 | 0.029 |
| ADGRD1 | -0.68 | -1.60 | 0.014 |
| NR0B1 | -0.76 | -1.70 | 0.003 |
| CACNA1D | -0.90 | -1.87 | 0.004 |
| LDHB | -1.17 | -2.26 | 0.006 |

FCs were calculated from the signal log ratios, which were calculated from *n* = 6 microarrays per group. Abbreviations: FC, fold change

**Supplemental Table 7** qPCR validation of microarray data

|  | FC | | *P*-value | |
| --- | --- | --- | --- | --- |
|  | Microarray | qPCR | Microarray | qPCR |
| *ANGPTL2* | -1.58 | -1.04 | 0.032 | 0.805 |
| C3AR1L | 1.55 | 1.10 | 0.007 | 0.487 |
| CCNB1 | -1.42 | -1.04 | 0.015 | 0.712 |
| FYB1 | 1.31 | 1.49 | 0.003 | 0.036 |
| HTR7 | 1.37 | 1.29 | 0.010 | 0.217 |
| KITLG | -1.32 | -1.06 | 0.020 | 0.772 |
| MYH1A | 1.63 | 2.21 | 0.041 | 0.087 |
| PHC1 | -1.39 | -1.04 | 0.020 | 0.753 |
| PLEK | 1.32 | 1.10 | 0.005 | 0.579 |
| PSTPIP2 | 1.47 | 1.62 | 0.006 | 0.005 |
| SMC2 | -1.40 | -1.07 | 0.026 | 0.719 |
| TBXAS1 | 1.41 | 1.26 | 0.011 | 0.385 |
| TOP2A | -1.45 | -1.54 | 0.008 | 0.101 |
| TOPAZ1 | 1.97 | 1.61 | 0.023 | 0.092 |
| TSHZ2 | -1.34 | -1.06 | 0.035 | 0.797 |
| WWTR1 | -1.39 | -1.06 | 0.025 | 0.666 |
| XCL1 | 1.58 | 1.40 | 0.013 | 0.080 |

Data are means for *n* = 6/group (microarray) and *n* = 12/group (qPCR)

**Supplemental Table 8** Plasma metabolites (µmol/L) of broilers fed diets with either 0% (PSA-0), 2.5% (PSA-2.5) or 5% (PSA-5.0) *P. sapidus* mycelium for 35 days.

|  | PSA-0 | PSA-2.5 | PSA-5.0 | FDR |
| --- | --- | --- | --- | --- |
| *Acylcarnitines* |  |  |  |  |
| C0 | 2.93 ± 1.18 | 2.49 ± 0.81 | 2.49 ± 0.52 | 0.805 |
| *Alkaloids* |  |  |  |  |
| Trigonelline | 1.57 ± 1.97 | 1.48 ± 2.28 | 1.18 ± 1.63 | 0.919 |
| *Amine Oxides* |  |  |  |  |
| TMAO | 1.2 ± 0.43 | 1.46 ± 0.24 | 1.53 ± 0.44 | 0.742 |
| *Aminoacids* |  |  |  |  |
| Alanine | 831 ± 164 | 872 ± 274 | 931 ± 294 | 0.961 |
| Arginine | 425 ± 136 | 372 ± 187 | 397 ± 137 | 0.901 |
| Asparagine | 218 ± 88 | 305 ± 85 | 290 ± 88 | 0.742 |
| Aspartic acid | 195 ± 86 | 125 ± 42 | 134 ± 58 | 0.742 |
| Cysteine | 70.4 ± 12.1 | 69.6 ± 13.2 | 69.1 ± 10.5 | 0.984 |
| Glutamine | 1573 ± 210 | 1712 ± 493 | 1499 ± 337 | 0.805 |
| Glutamic acid | 482 ± 194 | 392 ± 214 | 459 ± 258 | 0.903 |
| Glycine | 444 ± 100 | 431 ± 98 | 442 ± 45 | 0.960 |
| Histidine | 44.9 ± 9.3 | 43.4 ± 10.3 | 45.8 ± 10.8 | 0.960 |
| Isoleucine | 167 ± 90 | 177 ± 110 | 155 ± 54 | 0.978 |
| Leucine | 152 ± 49 | 180 ± 64 | 169 ± 39 | 0.787 |
| Lysine | 402 ± 142 | 417 ± 231 | 371 ± 118 | 0.960 |
| Methionine | 293 ± 159 | 302 ± 198 | 281 ± 147 | 0.973 |
| Phenylalanine | 119 ± 21 | 127 ± 26 | 121 ± 15 | 0.908 |
| Proline | 333 ± 60 | 365 ± 90 | 354 ± 61 | 0.903 |
| Serine | 523 ± 92 | 549 ± 111 | 607 ± 96 | 0.742 |
| Threonine | 347 ± 79 | 382 ± 143 | 360 ± 64 | 0.960 |
| Tryptophan | 84.6 ± 11.3 | 83.3 ± 8.7 | 86.8 ± 10.1 | 0.908 |
| Tyrosine | 267 ± 53 | 262 ± 52 | 247 ± 57 | 0.884 |
| Valine | 292 ± 111 | 313 ± 133 | 293 ± 73 | 0.965 |
| *Aminoacids Related* |  |  |  |  |
| 1-Met-His | 10.3 ± 3.2 | 7.11 ± 1.64 | 9.75 ± 2.66 | 0.742 |
| 3-Met-His | 8.16 ± 2.5 | 6.18 ± 1.21 | 8.3 ± 2.84 | 0.742 |
| AABA | 11 ± 1.8 | 10.4 ± 1.7 | 10.5 ± 2 | 0.908 |
| alpha-AAA | 2.29 ± 0.65 | 2.15 ± 0.58 | 2.19 ± 0.62 | 0.960 |
| Anserine | 46.4 ± 8.9 | 42.3 ± 9.2 | 41.2 ± 9.3 | 0.787 |
| Betaine | 225 ± 42 | 238 ± 37 | 266 ± 56 | 0.742 |
| Carnosine | 19.2 ± 5.4 | 14.9 ± 2.7 | 15.6 ± 4.8 | 0.742 |
| Citrulline | 5.2 ± 8.97 | 10.4 ± 13.4 | 3.72 ± 4.12 | 0.928 |
| Creatinine | 2.8 ± 1.51 | 3.56 ± 2.15 | 3.24 ± 1.87 | 0.991 |
| Cystine | 89.2 ± 29.5 | 86.3 ± 20.5 | 93.5 ± 31.4 | 0.980 |
| Homoarginie | 16.5 ± 10 | 13.6 ± 10.8 | 16.9 ± 8.8 | 0.786 |
| Homocysteine | 54.2 ± 10.6 | 55.4 ± 13.4 | 49.1 ± 8.4 | 0.805 |
| Met-SO | 6.34 ± 1.51 | 8.6 ± 5.37 | 9.04 ± 2.63 | 0.742 |
| Ornithine | 29.9 ± 14.6 | 47.6 ± 43.4 | 30.2 ± 11.3 | 0.878 |
| ProBetaine | 4.33 ± 0.98 | 4.56 ± 0.87 | 4.62 ± 1.5 | 0.960 |
| Sarcosine | 9.17 ± 2.04 | 10 ± 2.8 | 11.2 ± 2.2 | 0.742 |
| t4-OH-Pro | 160 ± 29 | 172 ± 29 | 157 ± 40 | 0.787 |
| Taurine | 136 ± 47 | 188 ± 106 | 153 ± 61 | 0.787 |
| *Bile Acids* |  |  |  |  |
| TCA | 0.966 ± 1.477 | 0.694 ± 0.461 | 0.797 ± 0.508 | 0.960 |
| TCDCA | 7.51 ± 6.52 | 8.1 ± 2.37 | 7.99 ± 3.49 | 0.787 |
| *Biogenic Amines* |  |  |  |  |
| beta-Alanine | 51.8 ± 24.4 | 58.3 ± 10.4 | 57.5 ± 18.7 | 0.787 |
| GABA | 3.02 ± 6.9 | 4.87 ± 11.65 | 2.27 ± 5.78 | 0.942 |
| Serotonin | 15.3 ± 23.4 | 6.23 ± 11.02 | 4.56 ± 7.62 | 0.965 |
| Spermidine | 1.21 ± 1.3 | 1.57 ± 2.32 | 1.23 ± 1.5 | 0.977 |
| *Carboxylic Acids* |  |  |  |  |
| AconAcid | 69.4 ± 26.1 | 63.5 ± 16.9 | 66 ± 27.9 | 0.960 |
| Lac | 6727 ± 2264 | 7039 ± 2617 | 7446 ± 2577 | 0.944 |
| OH-GlutAcid | 5.05 ± 2.29 | 4.92 ± 2.45 | 5.54 ± 4.64 | 0.988 |
| Suc | 44.9 ± 15.9 | 84.1 ± 115.3 | 53.6 ± 27.7 | 0.908 |
| *Ceramides* |  |  |  |  |
| Cer(d18:1/16:0) | 1.7 ± 0.22 | 1.99 ± 0.98 | 1.99 ± 0.29 | 0.768 |
| Cer(d18:1/22:0) | 2.18 ± 0.58 | 2.34 ± 1.21 | 2.5 ± 0.66 | 0.825 |
| Cer(d18:1/23:0) | 2.57 ± 0.76 | 2.71 ± 1.21 | 3.01 ± 0.87 | 0.787 |
| Cer(d18:1/24:0) | 3.86 ± 1.44 | 4.2 ± 2.04 | 4.58 ± 1.33 | 0.787 |
| Cer(d18:1/24:1) | 2.64 ± 0.55 | 3.03 ± 1.82 | 2.91 ± 1.09 | 0.960 |
| Cer(d18:1/25:0) | 3.83 ± 0.71 | 3.6 ± 1.4 | 3.93 ± 0.8 | 0.787 |
| CerP(d18:1/16:0) | 3.94 ± 0.86 | 3.95 ± 1.33 | 4.48 ± 1.11 | 0.786 |
| *Cholesterol Esters* |  |  |  |  |
| CE(14:0) | 4.85 ± 0.75 | 4.53 ± 1.06 | 4.67 ± 0.7 | 0.878 |
| CE(15:0) | 1.79 ± 0.3 | 1.94 ± 0.56 | 1.94 ± 0.36 | 0.903 |
| CE(15:1) | 0.647 ± 0.198 | 0.489 ± 0.207 | 0.628 ± 0.066 | 0.999 |
| CE(16:0) | 51.6 ± 55.7 | 83.9 ± 84.4 | 50 ± 61 | 0.812 |
| CE(16:1) | 20.6 ± 5 | 26 ± 9.6 | 24 ± 7 | 0.768 |
| CE(17:0) | 1.4 ± 0.23 | 1.71 ± 0.69 | 1.78 ± 0.41 | 0.742 |
| CE(18:0) | 10.7 ± 1.9 | 12.7 ± 5.8 | 10.7 ± 2.6 | 0.787 |
| CE(18:1) | 173 ± 33 | 201 ± 58 | 188 ± 46 | 0.805 |
| CE(18:2) | 1006 ± 143 | 1012 ± 192 | 1046 ± 205 | 0.961 |
| CE(18:3) | 71.4 ± 11.3 | 73.1 ± 15.7 | 75.3 ± 21.6 | 0.980 |
| CE(20:0) | 2.61 ± 2.32 | 3.61 ± 2.25 | 2.81 ± 2.32 | 0.958 |
| CE(20:1) | 1.39 ± 0.79 | 1.67 ± 0.48 | 1.4 ± 0.43 | 0.768 |
| CE(20:3) | 19.1 ± 3.6 | 20.2 ± 4.4 | 21.1 ± 4.7 | 0.884 |
| CE(20:4) | 66 ± 10.4 | 70.1 ± 27 | 71.3 ± 25.6 | 0.977 |
| CE(20:5) | 18.8 ± 3.5 | 19.8 ± 3.9 | 22.7 ± 8.1 | 0.786 |
| CE(22:5) | 4.41 ± 0.81 | 4.59 ± 2.15 | 4.4 ± 1.61 | 0.977 |
| CE(22:6) | 24.5 ± 3.7 | 25.8 ± 10.8 | 28.4 ± 10.9 | 0.908 |
| *Diacylglycerols* |  |  |  |  |
| DG(16:0_16:0) | 7.09 ± 1.51 | 7.31 ± 1.4 | 7.18 ± 0.96 | 0.961 |
| DG(16:0_18:1) | 1.9 ± 0.74 | 2.14 ± 0.91 | 2.51 ± 1.05 | 0.787 |
| DG(16:0_18:2) | 3.12 ± 0.89 | 2.73 ± 0.86 | 3.27 ± 0.98 | 0.786 |
| DG(16:1_18:1) | 2.36 ± 2.21 | 1.93 ± 1.86 | 2.54 ± 1.73 | 0.908 |
| DG(18:1_18:1) | 1.88 ± 0.57 | 2.3 ± 1.05 | 2.37 ± 0.9 | 0.787 |
| DG(18:1_18:2) | 10.2 ± 1.8 | 9.19 ± 1.24 | 9.81 ± 2.07 | 0.797 |
| DG(18:2_18:2) | 13.5 ± 3.7 | 11.2 ± 3.9 | 12.7 ± 3.9 | 0.773 |
| DG(18:2_18:3) | 1.28 ± 0.4 | 1.07 ± 0.42 | 1.23 ± 0.32 | 0.773 |
| *Fatty Acids* |  |  |  |  |
| FA(12:0) | 15.7 ± 1.8 | 16.4 ± 2.5 | 16.1 ± 2.6 | 0.944 |
| FA(14:0) | 1783 ± 443 | 1672 ± 369 | 1602 ± 336 | 0.860 |
| FA(16:0) | 409 ± 93 | 389 ± 94 | 395 ± 89 | 0.960 |
| FA(18:0) | 381 ± 174 | 338 ± 177 | 377 ± 161 | 0.942 |
| FA(18:1) | 67.5 ± 19.9 | 72.2 ± 23.2 | 60.4 ± 12.2 | 0.787 |
| FA(18:2) | 123 ± 73 | 131 ± 57 | 118 ± 62 | 0.938 |
| FA(20:1) | 2.14 ± 0.56 | 2.44 ± 0.49 | 2.18 ± 0.73 | 0.786 |
| FA(20:2) | 1.52 ± 0.36 | 1.57 ± 0.58 | 1.31 ± 0.53 | 0.773 |
| AA | 4.58 ± 1.85 | 4.84 ± 1.67 | 4.09 ± 1.88 | 0.848 |
| DHA | 1.53 ± 1.08 | 2.33 ± 0.82 | 1.55 ± 1.22 | 0.742 |
| *Glycosylceramides* |  |  |  |  |
| HexCer(d18:1/16:0) | 1.71 ± 0.38 | 1.8 ± 0.56 | 1.97 ± 0.61 | 0.825 |
| HexCer(d18:1/22:0) | 2.09 ± 0.47 | 1.94 ± 0.4 | 2.22 ± 0.37 | 0.755 |
| HexCer(d18:1/24:1) | 2.95 ± 0.46 | 3.1 ± 0.58 | 3.25 ± 0.55 | 0.787 |
| *Indoles Derivatives* |  |  |  |  |
| Indole sulfate | 1.15 ± 1.33 | 1.31 ± 0.8 | 1.09 ± 0.37 | 0.878 |
| Indole | 27.3 ± 21.4 | 14.2 ± 10.8 | 19.2 ± 10.8 | 0.908 |
| *Monoacylglycerols* |  |  |  |  |
| MG 18:1 | 9.06 ± 1.62 | 9.99 ± 2.59 | 10.7 ± 2.3 | 0.768 |
| MG 18:2 | 9.16 ± 2.65 | 8.01 ± 3.22 | 9.68 ± 3.68 | 0.787 |
| MG 18:3 | 1.33 ± 0.37 | 1.19 ± 0.53 | 1.49 ± 0.51 | 0.960 |
| MG 20:5 | 3.04 ± 1.31 | 2.69 ± 1.35 | 2.8 ± 1.35 | 0.960 |
| *Nucleobases Related* |  |  |  |  |
| Hypoxanthine | 8.2 ± 4.36 | 7.43 ± 2.7 | 6.93 ± 2.62 | 0.922 |
| Xanthine | 3.43 ± 1.84 | 3.54 ± 1.48 | 2.8 ± 1.2 | 0.785 |
| *Phosphatidic Acids* |  |  |  |  |
| LPA 18:2 | 2.27 ± 0.66 | 2.63 ± 0.71 | 2.75 ± 0.75 | 0.777 |
| PA 16:2_18:1 | 1.3 ± 0.36 | 1.16 ± 0.38 | 1.76 ± 0.91 | 0.787 |
| PA 18:0_18:1 | 2.41 ± 0.31 | 2.52 ± 0.56 | 2.58 ± 0.55 | 0.855 |
| PA 18:0_18:2 | 2.09 ± 0.23 | 2.21 ± 0.57 | 2.48 ± 0.43 | 0.742 |
| PA 18:1_18:2 | 0.967 ± 0.236 | 1.06 ± 0.37 | 1.15 ± 0.31 | 0.908 |
| PA 18:1_20:0 | 1.25 ± 0.22 | 1.68 ± 0.68 | 1.54 ± 0.3 | 0.742 |
| PA 18:2_20:0 | 4.38 ± 0.66 | 5.03 ± 1.22 | 4.99 ± 0.93 | 0.768 |
| PA 18:2_20:1 | 2.14 ± 0.27 | 2.4 ± 0.39 | 2.42 ± 0.51 | 0.757 |
| PA 18:2_22:0 | 1.17 ± 0.3 | 1.34 ± 0.54 | 1.3 ± 0.22 | 0.888 |
| PA 18:2_22:1 | 0.856 ± 0.289 | 0.987 ± 0.44 | 0.879 ± 0.115 | 0.773 |
| *Phosphatidylcholines* |  |  |  |  |
| lysoPC a C16:0 | 24.6 ± 4.1 | 25.9 ± 4.5 | 27.2 ± 4.6 | 0.787 |
| lysoPC a C18:0 | 18.7 ± 3 | 20.1 ± 4.2 | 21.4 ± 3.2 | 0.757 |
| lysoPC a C18:1 | 5.84 ± 0.88 | 7.39 ± 1.87 | 6.76 ± 1.39 | 0.742 |
| lysoPC a C18:2 | 14.2 ± 2 | 16.7 ± 3.5 | 16.5 ± 3 | 0.742 |
| lysoPC a C20:3 | 0.624 ± 0.153 | 0.828 ± 0.258 | 0.783 ± 0.248 | 0.742 |
| lysoPC a C20:4 | 2.7 ± 0.33 | 2.86 ± 0.5 | 2.81 ± 0.45 | 0.912 |
| PC aa C30:0 | 2.78 ± 0.29 | 2.97 ± 0.65 | 2.86 ± 0.47 | 0.908 |
| PC aa C32:0 | 29.2 ± 5.2 | 28.7 ± 6.1 | 30.4 ± 4.9 | 0.903 |
| PC aa C32:1 | 8.16 ± 1.93 | 10 ± 4.5 | 9.68 ± 3.24 | 0.805 |
| PC aa C32:2 | 2.91 ± 0.49 | 2.95 ± 0.44 | 3.03 ± 0.54 | 0.960 |
| PC aa C32:3 | 1.23 ± 0.27 | 1.2 ± 0.31 | 1.24 ± 0.23 | 0.960 |
| PC aa C34:1 | 153 ± 32 | 174 ± 54 | 173 ± 40 | 0.805 |
| PC aa C34:2 | 369 ± 47 | 352 ± 43 | 369 ± 57 | 0.896 |
| PC aa C34:3 | 21.9 ± 2.9 | 22.3 ± 3.6 | 23.1 ± 3.2 | 0.908 |
| PC aa C36:0 | 0.68 ± 0.306 | 0.49 ± 0.252 | 0.589 ± 0.192 | 0.775 |
| PC aa C36:1 | 85.5 ± 20.9 | 103 ± 42 | 99.6 ± 26.9 | 0.787 |
| PC aa C36:2 | 377 ± 58 | 364 ± 43 | 388 ± 69 | 0.903 |
| PC aa C36:3 | 105 ± 17 | 112 ± 20 | 111 ± 22 | 0.903 |
| PC aa C36:4 | 189 ± 27 | 175 ± 20 | 184 ± 29 | 0.848 |
| PC aa C36:5 | 16.3 ± 1.6 | 14.8 ± 1.7 | 17 ± 2.9 | 0.742 |
| PC aa C38:3 | 58.6 ± 14.4 | 64.4 ± 17.6 | 64.3 ± 16.3 | 0.894 |
| PC aa C38:4 | 242 ± 32 | 227 ± 34 | 234 ± 36 | 0.888 |
| PC aa C38:5 | 60 ± 9 | 57.7 ± 7.9 | 59.5 ± 11.6 | 0.960 |
| PC aa C38:6 | 40.5 ± 6.2 | 37.6 ± 5.9 | 40.4 ± 10 | 0.903 |
| PC aa C40:4 | 10.7 ± 1.7 | 10.5 ± 1.6 | 10.9 ± 1.6 | 0.958 |
| PC aa C40:5 | 16.7 ± 2.1 | 15.6 ± 3 | 16.3 ± 2.9 | 0.873 |
| PC aa C40:6 | 17.5 ± 2.5 | 16.6 ± 3.7 | 17.8 ± 3.3 | 0.903 |
| PC ae C32:1 | 2.22 ± 0.4 | 2.1 ± 0.38 | 2.46 ± 0.37 | 0.742 |
| PC ae C34:1 | 8.26 ± 1.1 | 7.76 ± 1.19 | 8.57 ± 0.83 | 0.742 |
| PC ae C34:2 | 13.5 ± 1.4 | 12.7 ± 1.9 | 14.3 ± 1.7 | 0.742 |
| PC ae C34:3 | 2.38 ± 0.27 | 2.34 ± 0.31 | 2.45 ± 0.3 | 0.903 |
| PC ae C36:1 | 2.23 ± 0.37 | 2.31 ± 0.42 | 2.4 ± 0.38 | 0.863 |
| PC ae C36:2 | 7.88 ± 0.77 | 7.38 ± 0.95 | 8.12 ± 0.89 | 0.742 |
| PC ae C36:3 | 5.32 ± 0.61 | 5.01 ± 0.71 | 5.3 ± 0.52 | 0.787 |
| PC ae C36:4 | 18.1 ± 2.1 | 15.8 ± 2.5 | 17.2 ± 1.5 | 0.742 |
| PC ae C36:5 | 3.19 ± 0.42 | 3.04 ± 0.59 | 3.06 ± 0.27 | 0.878 |
| PC ae C38:0 | 2.14 ± 0.35 | 1.99 ± 0.29 | 2.19 ± 0.61 | 0.903 |
| PC ae C38:2 | 3.22 ± 0.49 | 3.11 ± 0.52 | 3.43 ± 0.8 | 0.878 |
| PC ae C38:3 | 1.98 ± 0.3 | 1.99 ± 0.28 | 2.12 ± 0.4 | 0.867 |
| PC ae C38:4 | 13 ± 1.4 | 11.4 ± 1.7 | 12.8 ± 1.3 | 0.742 |
| PC ae C38:5 | 11.7 ± 1.3 | 10.7 ± 1.6 | 11.6 ± 1.2 | 0.742 |
| PC ae C38:6 | 2.71 ± 0.29 | 2.49 ± 0.43 | 2.67 ± 0.25 | 0.742 |
| PC ae C40:4 | 2.82 ± 0.3 | 2.69 ± 0.45 | 2.9 ± 0.34 | 0.787 |
| PC ae C40:5 | 2.84 ± 0.27 | 2.64 ± 0.41 | 2.9 ± 0.29 | 0.742 |
| *Phosphatidylethanolamines* |  |  |  |  |
| LPE 16:0 | 2.25 ± 0.62 | 2.5 ± 0.56 | 2.59 ± 0.64 | 0.787 |
| LPE 18:0 | 4.43 ± 1.11 | 4.97 ± 1.02 | 5.46 ± 0.84 | 0.742 |
| LPE 18:1 | 1.15 ± 0.22 | 1.58 ± 0.5 | 1.5 ± 0.39 | 0.742 |
| LPE 18:2 | 3.95 ± 0.71 | 4.91 ± 1.25 | 5.1 ± 1.05 | 0.742 |
| LPE 20:4 | 0.929 ± 0.14 | 1.12 ± 0.25 | 1.16 ± 0.17 | 0.742 |
| PE 34:1 | 5.69 ± 2.2 | 9.75 ± 6.27 | 8.86 ± 4.52 | 0.742 |
| PE 34:2 | 29.8 ± 5.9 | 34.1 ± 7.4 | 36.2 ± 8.5 | 0.742 |
| PE 34:3 | 1.78 ± 0.38 | 2.06 ± 0.52 | 2.29 ± 0.64 | 0.742 |
| PE 35:1 | 1.01 ± 0.26 | 1.85 ± 1.28 | 1.55 ± 0.45 | 0.742 |
| PE 35:2 | 3.6 ± 0.76 | 4.48 ± 1.63 | 4.42 ± 0.55 | 0.742 |
| PE 35:3 | 2.26 ± 0.31 | 2.72 ± 0.81 | 2.57 ± 0.33 | 0.742 |
| PE 36:1 | 5.83 ± 1.29 | 7.93 ± 3.07 | 7.45 ± 2.02 | 0.742 |
| PE 36:2 | 67.8 ± 12.9 | 77.1 ± 17.7 | 81.2 ± 17.1 | 0.742 |
| PE 36:3 | 16.4 ± 3 | 20.4 ± 4.7 | 20 ± 5.2 | 0.742 |
| PE 36:4 | 28.8 ± 3.9 | 31.4 ± 6.2 | 33 ± 6.5 | 0.768 |
| PE 36:5 | 3.6 ± 0.35 | 3.78 ± 0.87 | 4.32 ± 0.9 | 0.742 |
| PE 38:0 | 1.02 ± 0.19 | 1.06 ± 0.2 | 1.11 ± 0.22 | 0.882 |
| PE 38:2 | 2.1 ± 0.35 | 2.2 ± 0.55 | 2.43 ± 0.42 | 0.742 |
| PE 38:3 | 7.87 ± 2.09 | 9.53 ± 3.39 | 9.17 ± 2.94 | 0.787 |
| PE 38:4 | 102 ± 14 | 107 ± 19 | 111 ± 14 | 0.783 |
| PE 38:5 | 24.4 ± 4.3 | 27.2 ± 6 | 26.3 ± 5.3 | 0.825 |
| PE 38:6 | 24.3 ± 3.8 | 24.7 ± 4.6 | 26.1 ± 6.7 | 0.960 |
| PE 38:7 | 3.51 ± 0.48 | 3.65 ± 0.8 | 3.86 ± 0.78 | 0.878 |
| PE 40:4 | 3.83 ± 0.68 | 3.83 ± 0.73 | 3.93 ± 0.62 | 0.960 |
| PE 40:5 | 7.87 ± 1.21 | 7.9 ± 1.44 | 8.38 ± 1.66 | 0.908 |
| PE 40:6 | 10.1 ± 1.5 | 10.1 ± 2 | 11 ± 2.6 | 0.896 |
| PE 40:7 | 8.08 ± 1.22 | 8.46 ± 1.51 | 8.43 ± 1.72 | 0.960 |
| PE 40:8 | 2.86 ± 0.5 | 3.03 ± 0.75 | 2.95 ± 0.89 | 0.961 |
| PE 42:8 | 0.947 ± 0.234 | 0.991 ± 0.161 | 0.942 ± 0.254 | 0.912 |
| PE P-16:0/18:1 | 3.72 ± 0.75 | 3.83 ± 0.95 | 3.58 ± 0.68 | 0.955 |
| PE P-16:0/18:2 | 6.81 ± 1.88 | 7.47 ± 1.97 | 7.83 ± 1.85 | 0.787 |
| PE P-16:0/20:3 | 1.05 ± 0.2 | 0.904 ± 0.186 | 0.93 ± 0.186 | 0.742 |
| PE P-16:0/20:4 | 8.49 ± 1.73 | 7.73 ± 1.45 | 8.31 ± 1.34 | 0.805 |
| PE P-16:0/22:4 | 5.65 ± 0.98 | 5.23 ± 1.06 | 5.32 ± 0.76 | 0.863 |
| PE P-16:0/22:5 | 8.31 ± 1.85 | 7.74 ± 1.88 | 8.24 ± 1.87 | 0.915 |
| PE P-16:0/22:6 | 8.92 ± 2.6 | 9.11 ± 1.84 | 9.41 ± 2.46 | 0.960 |
| PE P-18:0/16:1 | 0.792 ± 0.401 | 1.09 ± 0.64 | 0.876 ± 0.515 | 0.755 |
| PE P-18:0/18:0 | 2.96 ± 1.22 | 2.6 ± 1.53 | 2.98 ± 0.96 | 0.742 |
| PE P-18:0/18:1 | 2.87 ± 0.83 | 4.17 ± 1.85 | 3.46 ± 0.91 | 0.742 |
| PE P-18:0/18:2 | 15.7 ± 4.1 | 18.7 ± 5 | 18.3 ± 3.8 | 0.742 |
| PE P-18:0/18:3 | 0.856 ± 0.219 | 0.963 ± 0.291 | 1.01 ± 0.2 | 0.768 |
| PE P-18:0/20:2 | 0.944 ± 0.23 | 1.25 ± 0.81 | 0.971 ± 0.279 | 0.787 |
| PE P-18:0/20:4 | 13.8 ± 2.8 | 14.5 ± 2.6 | 15.1 ± 2.2 | 0.796 |
| PE P-18:0/20:5 | 1.04 ± 0.22 | 1.08 ± 0.23 | 1.27 ± 0.35 | 0.742 |
| PE P-18:0/22:3 | 1.62 ± 0.64 | 2.04 ± 0.77 | 1.64 ± 0.65 | 0.787 |
| PE P-18:0/22:4 | 9.75 ± 2.06 | 10.1 ± 2.3 | 10.5 ± 2 | 0.908 |
| PE P-18:0/22:5 | 11.5 ± 3.2 | 11.4 ± 3 | 12.7 ± 3.5 | 0.896 |
| PE P-18:0/22:6 | 12.8 ± 4 | 14 ± 3.2 | 14.4 ± 3.8 | 0.825 |
| PE P-18:1/18:1 | 2.15 ± 0.53 | 2.45 ± 0.7 | 2.15 ± 0.38 | 0.787 |
| PE P-18:1/18:2 | 8.41 ± 2.43 | 9.2 ± 2.22 | 8.52 ± 1.7 | 0.881 |
| PE P-18:1/20:4 | 6.28 ± 1.67 | 5.68 ± 1.2 | 5.89 ± 0.97 | 0.888 |
| PE P-18:1/22:6 | 3.21 ± 1.09 | 3.14 ± 0.65 | 3.1 ± 0.78 | 0.988 |
| PE P-20:0/18:1 | 1.55 ± 0.93 | 0.844 ± 0.813 | 0.898 ± 0.793 | 0.742 |
| PE P-20:0/18:2 | 1.91 ± 0.43 | 2.18 ± 0.6 | 2.19 ± 0.41 | 0.768 |
| *Phosphatidylglycerols* |  |  |  |  |
| LPG 16:0 | 0.425 ± 0.289 | 0.421 ± 0.379 | 0.434 ± 0.181 | 0.965 |
| LPG 18:0 | 0.89 ± 0.368 | 0.681 ± 0.431 | 1.18 ± 0.42 | 0.965 |
| PG 16:0_18:2 | 0.812 ± 0.176 | 1.01 ± 0.46 | 0.95 ± 0.352 | 0.822 |
| PG 16:0_18:3 | 2.66 ± 0.34 | 2.65 ± 0.42 | 2.85 ± 0.41 | 0.787 |
| PG 16:1_18:1 | 1.57 ± 0.31 | 2 ± 0.79 | 1.88 ± 0.47 | 0.742 |
| PG 16:1_18:2 | 8.25 ± 1.52 | 8.5 ± 1.54 | 9.4 ± 1.74 | 0.742 |
| PG 16:1_20:4 | 2.08 ± 0.34 | 2.01 ± 0.42 | 2.2 ± 0.33 | 0.787 |
| PG 16:2_18:2 | 1.27 ± 0.12 | 1.52 ± 0.38 | 1.51 ± 0.25 | 0.742 |
| PG 17:0_18:2 | 0.267 ± 0.08 | 0.297 ± 0.175 | 0.362 ± 0.075 | 0.787 |
| PG 18:0_18:3 | 1.78 ± 0.29 | 1.9 ± 0.59 | 2.02 ± 0.32 | 0.768 |
| PG 18:1_18:1 | 1.1 ± 0.15 | 1.36 ± 0.54 | 1.33 ± 0.4 | 0.778 |
| PG 18:1_18:2 | 4.22 ± 0.77 | 4.42 ± 1.44 | 4.59 ± 0.84 | 0.883 |
| PG 18:1_20:3 | 0.968 ± 0.22 | 1.26 ± 0.51 | 1.16 ± 0.36 | 0.755 |
| PG 18:1_20:4 | 2.88 ± 0.69 | 3.73 ± 1.71 | 3.54 ± 0.97 | 0.768 |
| PG 18:1_22:4 | 1.36 ± 0.28 | 1.86 ± 0.84 | 1.72 ± 0.58 | 0.742 |
| PG 18:1_22:5 | 0.787 ± 0.128 | 0.958 ± 0.306 | 0.917 ± 0.205 | 0.749 |
| PG 18:2_20:3 | 2.96 ± 0.45 | 2.99 ± 0.45 | 3.17 ± 0.36 | 0.787 |
| PG 18:2_20:4 | 10 ± 1.4 | 9.91 ± 1.54 | 10.9 ± 1.4 | 0.768 |
| PG 18:2_22:3 | 2.87 ± 0.48 | 3.17 ± 0.96 | 3.28 ± 0.53 | 0.768 |
| PG 18:2_22:4 | 10.4 ± 1.6 | 11.4 ± 3.3 | 11.9 ± 1.8 | 0.768 |
| PG 20:4_20:4 | 1.51 ± 0.24 | 1.43 ± 0.25 | 1.56 ± 0.26 | 0.825 |
| PG 20:4_22:4 | 3.15 ± 0.49 | 3.09 ± 0.65 | 3.1 ± 0.46 | 0.976 |
| *Phosphatidylinositols* |  |  |  |  |
| PI 14:0_18:2 | 0.667 ± 0.137 | 0.805 ± 0.297 | 0.842 ± 0.234 | 0.860 |
| PI 15:0_16:0 | 5.06 ± 0.89 | 6.44 ± 2.1 | 6.22 ± 0.96 | 0.742 |
| PI 15:1_16:0 | 13 ± 1.9 | 14 ± 2.6 | 15.1 ± 2.1 | 0.742 |
| PI 16:0_16:0 | 0.445 ± 0.094 | 0.51 ± 0.184 | 0.549 ± 0.172 | 0.977 |
| PI 16:0_17:0 | 0.621 ± 0.205 | 0.673 ± 0.246 | 0.764 ± 0.163 | 0.878 |
| PI 16:0_17:1 | 1.74 ± 0.36 | 1.94 ± 0.46 | 1.9 ± 0.35 | 0.793 |
| PI 16:0_17:2 | 9.42 ± 1.7 | 11.7 ± 4.4 | 11.2 ± 2.7 | 0.742 |
| PI 16:0_18:1 | 2.03 ± 0.65 | 3.32 ± 2.06 | 2.67 ± 1.01 | 0.742 |
| PI 16:0_18:2 | 7.21 ± 0.97 | 7.92 ± 1.68 | 7.51 ± 1.32 | 0.832 |
| PI 16:0_20:0 | 1.67 ± 0.25 | 1.48 ± 0.48 | 1.69 ± 0.43 | 0.768 |
| PI 16:0_20:3 | 2.2 ± 0.51 | 3.07 ± 1.43 | 2.89 ± 0.97 | 0.742 |
| PI 16:0_20:4 | 3.68 ± 0.85 | 4.41 ± 1.58 | 4.33 ± 1.22 | 0.786 |
| PI 16:1_18:1 | 0.952 ± 0.312 | 1.2 ± 0.39 | 1.16 ± 0.38 | 0.742 |
| PI 16:1_18:2 | 3.69 ± 0.74 | 4.07 ± 0.85 | 4.42 ± 0.79 | 0.742 |
| PI 17:1_18:1 | 7.63 ± 1.44 | 10.2 ± 4.9 | 9.16 ± 2.82 | 0.755 |
| PI 17:1_18:2 | 48 ± 6.7 | 51.7 ± 14.6 | 54.8 ± 7.7 | 0.752 |
| PI 18:0_18:1 | 2.43 ± 0.51 | 3.08 ± 1.09 | 2.74 ± 0.62 | 0.742 |
| PI 18:0_18:2 | 24.7 ± 2.9 | 26.8 ± 5.6 | 26.1 ± 4.6 | 0.867 |
| PI 18:0_18:3 | 1.08 ± 0.2 | 1.22 ± 0.51 | 1.2 ± 0.22 | 0.867 |
| PI 18:0_20:0 | 1.27 ± 0.38 | 1.13 ± 0.43 | 1.44 ± 0.5 | 0.773 |
| PI 18:0_20:3 | 5.83 ± 1.04 | 6.75 ± 2.1 | 6.59 ± 1.96 | 0.805 |
| PI 18:0_20:4 | 17.6 ± 2.8 | 18.3 ± 3.4 | 20.4 ± 5.6 | 0.786 |
| PI 18:0_22:0 | 0.724 ± 0.13 | 0.935 ± 0.354 | 1.02 ± 0.25 | 0.742 |
| PI 18:1_18:1 | 1.25 ± 0.21 | 1.82 ± 0.67 | 1.57 ± 0.55 | 0.742 |
| PI 18:1_18:2 | 2.8 ± 0.33 | 3.35 ± 0.45 | 3.14 ± 0.53 | 0.742 |
| PI 18:1_20:3 | 0.569 ± 0.129 | 0.846 ± 0.383 | 0.77 ± 0.36 | 0.958 |
| PI 18:2_18:3 | 0.733 ± 0.272 | 0.826 ± 0.393 | 1.15 ± 0.23 | 0.960 |
| PI 18:2_20:5 | 0.6 ± 0.223 | 0.746 ± 0.261 | 0.815 ± 0.256 | 0.742 |
| *Phosphatidylserines* |  |  |  |  |
| PS 38:5 | 1.3 ± 0.19 | 1.32 ± 0.35 | 1.4 ± 0.26 | 0.887 |
| *Sphingoid Bases* |  |  |  |  |
| C14 Sphingosine-1-Phosphate | 2.13 ± 0.66 | 2.23 ± 0.7 | 2.11 ± 0.66 | 0.961 |
| C17 Sphingosine-1-Phosphate | 0.441 ± 0.117 | 0.552 ± 0.226 | 0.501 ± 0.09 | 0.742 |
| C18 Sphingosine-1-Phosphate | 0.513 ± 0.397 | 0.455 ± 0.335 | 0.415 ± 0.228 | 0.965 |
| *Sphingomyelins* |  |  |  |  |
| SM C16:0 | 130 ± 13 | 137 ± 24 | 140 ± 9 | 0.768 |
| SM C18:0 | 11.2 ± 1.1 | 11.5 ± 1.6 | 12.5 ± 1.1 | 0.742 |
| SM C22:3 | 0.206 ± 0.119 | 0.248 ± 0.156 | 0.277 ± 0.166 | 0.878 |
| SM (OH) C22:1 | 2.06 ± 0.29 | 2.18 ± 0.21 | 2.38 ± 0.29 | 0.742 |
| SM C24:0 | 9.04 ± 1.47 | 10.1 ± 2.2 | 10.5 ± 1.7 | 0.742 |
| SM C24:1 | 6.22 ± 1.92 | 5.85 ± 1.46 | 7.14 ± 1.4 | 0.742 |
| *Sugars* |  |  |  |  |
| H1 | 14240 ± 920 | 14240 ± 700 | 14510 ± 710 | 0.895 |
| *Triglyceride* |  |  |  |  |
| TG(14:0_34:0) | 0.466 ± 0.37 | 1.17 ± 0.91 | 1.12 ± 0.89 | 0.768 |
| TG(14:0_34:1) | 2.56 ± 2.39 | 4.37 ± 3.67 | 5.84 ± 2.61 | 0.742 |
| TG(14:0_34:2) | 3.11 ± 1.37 | 3.22 ± 1.91 | 4.44 ± 1.87 | 0.742 |
| TG(14:0_34:3) | 0.31 ± 0.177 | 0.303 ± 0.208 | 0.51 ± 0.28 | 0.742 |
| TG(14:0_36:1) | 2.66 ± 0.59 | 3.38 ± 1.48 | 3.66 ± 1.03 | 0.742 |
| TG(14:0_36:2) | 2.02 ± 1.18 | 2.48 ± 1.99 | 3.14 ± 1.21 | 0.742 |
| TG(14:0_36:3) | 2.5 ± 0.95 | 2.05 ± 1.19 | 2.76 ± 1.59 | 0.787 |
| TG(14:0_36:4) | 1.58 ± 0.52 | 1.31 ± 0.63 | 1.82 ± 0.95 | 0.755 |
| TG(16:0_30:2) | 0.261 ± 0.269 | 0.348 ± 0.22 | 0.466 ± 0.287 | 0.742 |
| TG(16:0_32:0) | 2.75 ± 2.55 | 10.5 ± 13.2 | 4.37 ± 5.26 | 0.825 |
| TG(16:0_32:1) | 5.39 ± 7.27 | 10.2 ± 8.1 | 12.4 ± 8.7 | 0.742 |
| TG(16:0_32:2) | 3.92 ± 3.18 | 5 ± 3.4 | 6.45 ± 3 | 0.742 |
| TG(16:0_32:3) | 0.856 ± 0.56 | 0.978 ± 0.477 | 1.23 ± 0.52 | 0.742 |
| TG(16:0_33:1) | 0.338 ± 0.239 | 0.499 ± 0.407 | 0.689 ± 0.489 | 0.825 |
| TG(16:0_33:2) | 0.548 ± 0.239 | 0.652 ± 0.284 | 0.852 ± 0.339 | 0.742 |
| TG(16:0_34:0) | 10.7 ± 11.9 | 23.5 ± 35.6 | 16.3 ± 14.2 | 0.908 |
| TG(16:0_34:1) | 48 ± 34.6 | 69.9 ± 46.1 | 91.4 ± 46.7 | 0.742 |
| TG(16:0_34:2) | 64 ± 30 | 78.1 ± 32.1 | 99.1 ± 46.1 | 0.742 |
| TG(16:0_34:3) | 11.3 ± 5.9 | 13.6 ± 6.4 | 16.8 ± 7.5 | 0.742 |
| TG(16:0_34:4) | 1.73 ± 0.75 | 1.76 ± 0.52 | 2.14 ± 1.1 | 0.805 |
| TG(16:0_35:1) | 0.866 ± 0.454 | 1.27 ± 0.88 | 1.5 ± 0.73 | 0.764 |
| TG(16:0_35:2) | 1.15 ± 0.39 | 1.37 ± 0.63 | 1.85 ± 0.75 | 0.742 |
| TG(16:0_35:3) | 0.719 ± 0.242 | 0.678 ± 0.216 | 0.916 ± 0.374 | 0.742 |
| TG(16:0_36:2) | 107 ± 45 | 136 ± 63 | 155 ± 56 | 0.742 |
| TG(16:0_36:3) | 144 ± 37 | 149 ± 44 | 177 ± 64 | 0.768 |
| TG(16:0_36:4) | 90.2 ± 23.4 | 88.6 ± 31.9 | 115 ± 57 | 0.768 |
| TG(16:0_36:5) | 15.7 ± 4.7 | 16.1 ± 6.4 | 20.9 ± 11.5 | 0.768 |
| TG(16:0_36:6) | 1.11 ± 0.38 | 0.98 ± 0.554 | 1.38 ± 0.83 | 0.742 |
| TG(16:0_38:1) | 1.01 ± 0.4 | 1.27 ± 0.34 | 1.03 ± 0.6 | 0.960 |
| TG(16:0_38:2) | 2.27 ± 0.95 | 2.84 ± 1.07 | 3.15 ± 1.02 | 0.742 |
| TG(16:0_38:3) | 5.61 ± 1.31 | 5.4 ± 1.62 | 6.31 ± 1.87 | 0.787 |
| TG(16:0_38:4) | 4.74 ± 1.47 | 4.55 ± 1.3 | 5.45 ± 1.86 | 0.795 |
| TG(16:0_38:5) | 4.24 ± 0.85 | 4.44 ± 1.22 | 5.05 ± 1.74 | 0.824 |
| TG(16:0_38:6) | 2.44 ± 0.53 | 1.91 ± 0.52 | 2.41 ± 0.96 | 0.742 |
| TG(16:0_38:7) | 0.546 ± 0.247 | 0.657 ± 0.229 | 0.63 ± 0.364 | 0.960 |
| TG(16:0_40:6) | 4.52 ± 1.45 | 3.58 ± 1.12 | 3.83 ± 1.5 | 0.764 |
| TG(16:0_40:7) | 2.23 ± 0.87 | 1.59 ± 0.43 | 1.75 ± 0.81 | 0.742 |
| TG(16:1_32:0) | 1.49 ± 2.51 | 2.98 ± 2.9 | 3.9 ± 3.61 | 0.742 |
| TG(16:1_32:1) | 1.16 ± 2.09 | 1.77 ± 2.3 | 2.31 ± 2.49 | 0.764 |
| TG(16:1_32:2) | 0.288 ± 0.306 | 0.51 ± 0.462 | 0.598 ± 0.32 | 0.742 |
| TG(16:1_34:0) | 2.47 ± 2.82 b | 5.02 ± 4.22 ab | 6.07 ± 3.01 a | 0.742 |
| TG(16:1_34:1) | 9.57 ± 13.46 | 17 ± 16.5 | 18.1 ± 15.8 | 0.742 |
| TG(16:1_34:2) | 8.01 ± 5.43 | 9.92 ± 5.71 | 11.6 ± 6.1 | 0.768 |
| TG(16:1_34:3) | 1.07 ± 0.74 | 1.49 ± 0.79 | 1.72 ± 0.84 | 0.742 |
| TG(16:1_36:1) | 2.66 ± 1.74 | 3.91 ± 3 | 4.52 ± 2.27 | 0.742 |
| TG(16:1_36:2) | 6.77 ± 3.8 | 9.61 ± 6.63 | 9.64 ± 3.97 | 0.755 |
| TG(16:1_36:3) | 8.15 ± 2.05 | 8.48 ± 2.86 | 9.11 ± 3.01 | 0.903 |
| TG(16:1_36:4) | 4.59 ± 1.14 | 4.74 ± 1.27 | 5.71 ± 2.71 | 0.778 |
| TG(16:1_36:5) | 0.987 ± 0.338 | 1.03 ± 0.25 | 1.18 ± 0.6 | 0.884 |
| TG(16:1_38:3) | 0.53 ± 0.145 | 0.517 ± 0.14 | 0.519 ± 0.144 | 0.960 |
| TG(16:1_38:4) | 0.798 ± 0.239 | 0.941 ± 0.281 | 0.929 ± 0.392 | 0.855 |
| TG(17:0_34:1) | 0.527 ± 0.254 | 0.795 ± 0.355 | 0.97 ± 0.423 | 0.742 |
| TG(17:0_34:2) | 0.989 ± 0.233 | 1.09 ± 0.24 | 1.41 ± 0.52 | 0.742 |
| TG(17:0_36:3) | 1.59 ± 0.29 | 1.58 ± 0.42 | 1.8 ± 0.61 | 0.860 |
| TG(17:0_36:4) | 1.12 ± 0.33 | 1.23 ± 0.43 | 1.54 ± 0.78 | 0.755 |
| TG(17:1_34:1) | 0.409 ± 0.288 | 0.788 ± 0.435 | 0.718 ± 0.513 | 0.742 |
| TG(17:1_34:2) | 0.607 ± 0.187 | 0.666 ± 0.207 | 0.825 ± 0.315 | 0.742 |
| TG(17:2_34:2) | 0.572 ± 0.19 | 0.548 ± 0.169 | 0.592 ± 0.236 | 0.742 |
| TG(17:2_36:2) | 0.379 ± 0.079 | 0.482 ± 0.095 | 0.497 ± 0.086 | 0.786 |
| TG(17:2_36:3) | 0.689 ± 0.352 | 0.664 ± 0.412 | 0.982 ± 0.677 | 0.786 |
| TG(17:2_36:4) | 0.998 ± 0.284 | 1.06 ± 0.37 | 1.23 ± 0.65 | 0.742 |
| TG(18:0_30:0) | 0.729 ± 0.715 | 1.22 ± 0.76 | 1.46 ± 1.66 | 0.855 |
| TG(18:0_30:1) | 0.232 ± 0.244 | 0.381 ± 0.257 | 0.395 ± 0.344 | 0.742 |
| TG(18:0_32:0) | 1.42 ± 1.48 | 5.69 ± 7.04 | 2.15 ± 3.33 | 0.965 |
| TG(18:0_32:1) | 2.3 ± 1.95 | 3.64 ± 2.6 | 4.85 ± 2.42 | 0.742 |
| TG(18:0_32:2) | 1.11 ± 0.41 | 1.15 ± 0.67 | 1.55 ± 0.47 | 0.742 |
| TG(18:0_34:2) | 30 ± 11.8 | 34.5 ± 17.2 | 43.1 ± 17.6 | 0.755 |
| TG(18:0_34:3) | 3.63 ± 1.31 | 4.14 ± 1.64 | 5.15 ± 2.02 | 0.742 |
| TG(18:0_36:1) | 4.49 ± 1.11 | 5.43 ± 2.02 | 9.8 ± 12.15 | 0.742 |
| TG(18:0_36:2) | 15.4 ± 3.2 | 16.7 ± 7.5 | 20.7 ± 6 | 0.742 |
| TG(18:0_36:3) | 23.1 ± 5.2 | 23.3 ± 7.4 | 29.7 ± 11.7 | 0.742 |
| TG(18:0_36:4) | 23 ± 5.9 | 22.9 ± 8.9 | 27.8 ± 14.6 | 0.805 |
| TG(18:0_36:5) | 4.02 ± 0.91 | 4.02 ± 1.81 | 5.01 ± 2.3 | 0.768 |
| TG(18:0_38:6) | 1.14 ± 0.42 | 1 ± 0.31 | 1.06 ± 0.44 | 0.921 |
| TG(18:0_38:7) | 0.344 ± 0.164 | 0.354 ± 0.114 | 0.404 ± 0.164 | 0.742 |
| TG(18:1_30:0) | 2.1 ± 1.89 | 3.07 ± 3.18 | 4.46 ± 2.83 | 0.742 |
| TG(18:1_30:1) | 0.787 ± 0.999 | 1.29 ± 1.6 | 1.69 ± 1.49 | 0.742 |
| TG(18:1_32:0) | 19.9 ± 16.4 | 32.9 ± 22 | 43.1 ± 21.2 | 0.742 |
| TG(18:1_32:1) | 8.93 ± 10.95 | 16.5 ± 16.1 | 18 ± 14.4 | 0.742 |
| TG(18:1_32:2) | 3.05 ± 1.51 | 3.98 ± 2.5 | 4.22 ± 1.57 | 0.768 |
| TG(18:1_33:0) | 0.649 ± 0.323 | 0.823 ± 0.455 | 1.03 ± 0.48 | 0.757 |
| TG(18:1_33:1) | 0.834 ± 0.532 | 0.904 ± 0.795 | 1.3 ± 0.81 | 0.752 |
| TG(18:1_33:2) | 1.01 ± 0.32 | 1.08 ± 0.3 | 1.3 ± 0.58 | 0.742 |
| TG(18:1_34:1) | 135 ± 84 | 186 ± 120 | 206 ± 96 | 0.742 |
| TG(18:1_34:2) | 143 ± 40 | 149 ± 43 | 178 ± 64 | 0.768 |
| TG(18:1_34:3) | 18.5 ± 4.9 | 19.9 ± 5.5 | 23.8 ± 10.4 | 0.755 |
| TG(18:1_34:4) | 2.46 ± 0.66 | 2.51 ± 0.67 | 2.96 ± 1.2 | 0.787 |
| TG(18:1_35:2) | 1.64 ± 0.33 | 1.62 ± 0.45 | 2.04 ± 0.77 | 0.742 |
| TG(18:1_36:0) | 2.81 ± 1.5 | 3.57 ± 2.37 | 5.81 ± 5.67 | 0.742 |
| TG(18:1_36:1) | 20.1 ± 7.3 | 26.4 ± 13.8 | 31.9 ± 12.7 | 0.742 |
| TG(18:1_36:2) | 57.4 ± 10.7 | 64.1 ± 25 | 73.8 ± 24.3 | 0.742 |
| TG(18:1_36:3) | 106 ± 22 | 111 ± 41 | 134 ± 64 | 0.777 |
| TG(18:1_36:4) | 103 ± 31 | 105 ± 43 | 138 ± 81 | 0.768 |
| TG(18:1_36:5) | 24.9 ± 7.6 | 26.3 ± 10.8 | 33.2 ± 19.7 | 0.777 |
| TG(18:1_36:6) | 2.36 ± 0.7 | 2.56 ± 1.09 | 3.2 ± 2.06 | 0.787 |
| TG(18:1_38:5) | 6.23 ± 1.37 | 5.89 ± 1.39 | 5.86 ± 1.92 | 0.912 |
| TG(18:1_38:6) | 3.25 ± 0.91 | 2.66 ± 0.67 | 2.82 ± 0.98 | 0.768 |
| TG(18:1_38:7) | 0.883 ± 0.439 | 0.607 ± 0.19 | 0.757 ± 0.336 | 0.768 |
| TG(18:2_30:0) | 2.16 ± 1.31 | 2.47 ± 1.46 | 3.41 ± 1.73 | 0.742 |
| TG(18:2_32:0) | 27.1 ± 10.2 | 29.4 ± 14.9 | 40.5 ± 18.7 | 0.742 |
| TG(18:2_32:1) | 6.56 ± 3.84 | 7.63 ± 3.78 | 9.67 ± 4.68 | 0.742 |
| TG(18:2_32:2) | 3.65 ± 1.03 | 3.35 ± 1.28 | 4.12 ± 1.98 | 0.787 |
| TG(18:2_33:0) | 0.799 ± 0.204 | 0.843 ± 0.358 | 1.1 ± 0.45 | 0.742 |
| TG(18:2_33:1) | 1.12 ± 0.36 | 1.24 ± 0.37 | 1.52 ± 0.69 | 0.742 |
| TG(18:2_33:2) | 1.61 ± 0.51 | 1.69 ± 0.54 | 2.33 ± 1.22 | 0.742 |
| TG(18:2_34:0) | 47.6 ± 16.6 | 54.8 ± 27.6 | 74.8 ± 32.1 | 0.742 |
| TG(18:2_34:1) | 119 ± 34 | 119 ± 33 | 148 ± 51 | 0.742 |
| TG(18:2_34:2) | 135 ± 37 | 137 ± 49 | 171 ± 80 | 0.773 |
| TG(18:2_34:3) | 19 ± 5.2 | 19.2 ± 6 | 24.3 ± 13.4 | 0.785 |
| TG(18:2_34:4) | 4.04 ± 1.57 | 3.96 ± 1.56 | 4.73 ± 2.74 | 0.903 |
| TG(18:2_35:1) | 1.94 ± 0.61 | 2.02 ± 0.61 | 2.61 ± 1.33 | 0.742 |
| TG(18:2_35:2) | 1.91 ± 0.45 | 1.99 ± 0.59 | 2.46 ± 0.97 | 0.742 |
| TG(18:2_35:3) | 1.07 ± 0.28 | 1.16 ± 0.38 | 1.51 ± 0.89 | 0.742 |
| TG(18:2_36:0) | 4.8 ± 1.9 | 5.07 ± 3.16 | 6.67 ± 2.79 | 0.742 |
| TG(18:2_36:1) | 23.6 ± 5.2 | 24.2 ± 8 | 30.3 ± 13.1 | 0.742 |
| TG(18:2_36:2) | 83.8 ± 20.3 | 82.3 ± 27.5 | 100 ± 41 | 0.777 |
| TG(18:2_36:3) | 174 ± 55 | 171 ± 70 | 217 ± 136 | 0.787 |
| TG(18:2_36:4) | 161 ± 44 | 164 ± 76 | 200 ± 117 | 0.805 |
| TG(18:2_36:5) | 33.2 ± 10.6 | 32.7 ± 15.3 | 41.5 ± 24.8 | 0.787 |
| TG(18:2_38:4) | 4.23 ± 0.93 | 3.25 ± 0.92 | 4.13 ± 1.38 | 0.742 |
| TG(18:2_38:5) | 4.71 ± 1.42 | 3.57 ± 0.9 | 4.17 ± 1.48 | 0.742 |
| TG(18:2_38:6) | 3.04 ± 1.1 | 2.5 ± 0.97 | 2.83 ± 1.38 | 0.878 |
| TG(18:3_32:0) | 2.43 ± 1.13 | 2.62 ± 1.41 | 3.78 ± 1.69 | 0.742 |
| TG(18:3_32:1) | 0.617 ± 0.437 | 0.806 ± 0.388 | 1.04 ± 0.49 | 0.742 |
| TG(18:3_34:0) | 4.84 ± 2.46 | 5.75 ± 3.04 | 7.69 ± 3.41 | 0.742 |
| TG(18:3_34:1) | 12.8 ± 4.1 | 13.5 ± 4.8 | 17.6 ± 8.2 | 0.742 |
| TG(18:3_34:2) | 15.3 ± 4.5 | 15.9 ± 5.6 | 20.6 ± 11.4 | 0.768 |
| TG(18:3_34:3) | 2.46 ± 0.79 | 2.41 ± 0.8 | 3.07 ± 1.77 | 0.787 |
| TG(18:3_36:1) | 3.75 ± 0.94 | 4.4 ± 1.61 | 5.55 ± 3.24 | 0.742 |
| TG(18:3_36:2) | 13.4 ± 3.8 | 13.1 ± 5.1 | 17.4 ± 9.2 | 0.755 |
| TG(18:3_36:3) | 25.5 ± 7.1 | 26 ± 10.5 | 34.4 ± 19.1 | 0.742 |
| TG(18:3_36:4) | 20.4 ± 6.5 | 21.3 ± 9.5 | 27.4 ± 17.5 | 0.786 |
| TG(18:3_38:6) | 0.612 ± 0.112 | 0.479 ± 0.148 | 0.603 ± 0.224 | 0.942 |
| TG(20:0_32:4) | 0.752 ± 0.202 | 0.687 ± 0.201 | 0.853 ± 0.463 | 0.742 |
| TG(20:0_34:1) | 0.783 ± 0.683 | 0.683 ± 0.505 | 0.932 ± 0.389 | 0.768 |
| TG(20:1_31:0) | 1.3 ± 0.48 | 1.28 ± 0.46 | 1.17 ± 0.36 | 0.928 |
| TG(20:1_34:1) | 1.16 ± 0.64 | 1.51 ± 1 | 1.64 ± 0.82 | 0.768 |
| TG(20:1_34:2) | 1.31 ± 0.33 | 1.54 ± 0.58 | 1.68 ± 0.8 | 0.805 |
| TG(20:2_32:0) | 0.604 ± 0.31 | 0.677 ± 0.287 | 0.819 ± 0.249 | 0.742 |
| TG(20:2_34:1) | 2.72 ± 0.83 | 2.71 ± 0.92 | 2.79 ± 0.77 | 0.965 |
| TG(20:2_34:2) | 1.68 ± 0.42 | 1.5 ± 0.5 | 2.03 ± 0.79 | 0.742 |
| TG(20:3_32:0) | 0.335 ± 0.196 | 0.568 ± 0.252 | 0.623 ± 0.247 | 0.742 |
| TG(20:3_34:0) | 1.32 ± 0.58 | 1.7 ± 0.75 | 1.74 ± 0.74 | 0.863 |
| TG(20:3_34:1) | 2.39 ± 0.81 | 3 ± 1.45 | 3.21 ± 0.99 | 0.742 |
| TG(20:3_34:2) | 1.82 ± 0.42 | 1.96 ± 0.66 | 2.33 ± 0.7 | 0.742 |
| TG(20:3_34:3) | 0.779 ± 0.273 | 0.73 ± 0.227 | 0.888 ± 0.376 | 0.742 |
| TG(20:3_36:3) | 1.84 ± 0.41 | 1.74 ± 0.55 | 1.71 ± 0.58 | 0.908 |
| TG(20:3_36:4) | 1.43 ± 0.38 | 1.31 ± 0.43 | 1.45 ± 0.59 | 0.908 |
| TG(20:4_32:0) | 1.11 ± 0.52 | 1.35 ± 0.63 | 1.78 ± 0.75 | 0.768 |
| TG(20:4_32:1) | 0.459 ± 0.337 | 0.587 ± 0.352 | 0.807 ± 0.419 | 0.742 |
| TG(20:4_34:0) | 3.83 ± 1.85 | 3.72 ± 2.11 | 5.1 ± 2.46 | 0.777 |
| TG(20:4_34:1) | 7.33 ± 1.56 | 8.14 ± 2.04 | 8.62 ± 2.86 | 0.869 |
| TG(20:4_34:2) | 7.15 ± 2.03 | 6.28 ± 1.62 | 6.97 ± 2.64 | 0.908 |
| TG(20:4_34:3) | 1.27 ± 0.41 | 1.08 ± 0.3 | 1.21 ± 0.51 | 0.903 |
| TG(20:4_36:2) | 7.22 ± 1.99 | 6.89 ± 1.92 | 6.74 ± 2.45 | 0.942 |
| TG(20:4_36:3) | 8.52 ± 3.04 | 6.74 ± 2.27 | 7.36 ± 2.87 | 0.787 |
| TG(20:4_36:4) | 5.87 ± 2.09 | 5.02 ± 2.03 | 5.34 ± 2.18 | 0.896 |
| TG(20:4_36:5) | 1.48 ± 0.5 | 1.22 ± 0.51 | 1.39 ± 0.59 | 0.863 |
| TG(20:5_34:0) | 0.634 ± 0.276 | 1.05 ± 0.2 | 1.24 ± 0.21 | 0.968 |
| TG(20:5_34:1) | 1.24 ± 0.3 | 1.3 ± 0.54 | 1.59 ± 0.67 | 0.773 |
| TG(20:5_34:2) | 1.03 ± 0.35 | 0.781 ± 0.252 | 1.12 ± 0.66 | 0.787 |
| TG(20:5_36:2) | 1.05 ± 0.46 | 0.799 ± 0.298 | 0.937 ± 0.557 | 0.787 |
| TG(20:5_36:3) | 1.41 ± 0.76 | 0.949 ± 0.278 | 1.37 ± 0.78 | 0.742 |
| TG(22:4_32:0) | 0.67 ± 0.316 | 0.593 ± 0.184 | 0.771 ± 0.359 | 0.965 |
| TG(22:4_34:2) | 1.27 ± 0.4 | 1.02 ± 0.33 | 1.22 ± 0.55 | 0.787 |
| TG(22:5_32:0) | 0.723 ± 0.261 | 0.696 ± 0.302 | 0.879 ± 0.375 | 0.742 |
| TG(22:5_34:1) | 5.43 ± 1.51 | 4.6 ± 1.2 | 4.55 ± 1.68 | 0.768 |
| TG(22:5_34:2) | 2.48 ± 0.77 | 1.85 ± 0.55 | 2.07 ± 1.02 | 0.742 |
| TG(22:6_32:0) | 0.47 ± 0.136 | 0.593 ± 0.155 | 0.668 ± 0.321 | 0.960 |
| TG(22:6_34:1) | 1.94 ± 0.57 | 2.09 ± 0.55 | 2.04 ± 0.84 | 0.960 |
| TG(22:6_34:2) | 1.66 ± 0.57 | 1.54 ± 0.55 | 1.56 ± 0.71 | 0.958 |
| TG(22:6_34:3) | 0.355 ± 0.105 | 0.379 ± 0.138 | 0.379 ± 0.121 | 0.867 |
| *Vitamins & Cofactors* |  |  |  |  |
| Choline | 45.9 ± 43.8 | 56.1 ± 70.6 | 39.1 ± 38.2 | 0.908 |

Data are means ± SD for *n* = 10 broilers/group.

Abbreviations: 1‑Met‑His, 1‑methylhistidine; 3‑Met‑His, 3‑methylhistidine; AA, arachidonic acid; AABA, α‑Aminobutyric acid; alpha-AAA, alpha-amonoadipic acid; AconAcid, aconitic acid; CE, cholesterol ester; Cer, ceramide; DG, diacylglycerol; DHA, docosahexaenoic acid; FA, fatty acid; GABA, gamma-aminobutyric acid; HexCer, hexosylceraminde; Lac, laccaic acid; LPA, lysophosphatic acid; LPE, lysophosphatidylethanolamine; LPG, lysophosphatidylglycerol; lysoPC a, lysophosphatidylcholine; Met‑SO, methioninesulfoxid; MG, monoacylglycerols; OH-GlutAcid, hydroxyglutamic acid; PC aa, diacylphosphatidylcholine; PA, phosphatic acid; PC ae, acyl‑alkyl‑phosphatidylcholine; PE, phosphatidylethanolamine; PE P, phosphatidylethanolamine plasmenyl; PG, phosphatidylglycerol; PI, phosphatidylinositol; PS, phosphatidylserine; SM, sphingolipid; Suc, succinate; t4-OH-Pro, trans-4-hydroxoproline; TCA, taurocholic acid; TCDCA, taurochenodeoxycholic acid; TG, triglyceride; TMAO, trimethylamine *N*‑oxide
